# Fibro-adipogenic progenitors in physiological adipogenesis and intermuscular adipose tissue remodeling

**DOI:** 10.1016/j.mam.2024.101277

**Published:** 2024-05-23

**Authors:** Marcelo Flores-Opazo, Daniel Kopinke, Françoise Helmbacher, Rodrigo Fernández-Verdejo, Mauro Tuñón-Suárez, Gordon S. Lynch, Osvaldo Contreras

**Affiliations:** a Institute of Health Sciences, Universidad de O’Higgins, Rancagua, Chile; b Department of Pharmacology and Therapeutics, University of Florida, Gainesville, 32610, FL, USA; c Myology Institute, University of Florida College of Medicine, Gainesville, FL, USA; d Aix Marseille Univ, CNRS, IBDM, UMR, 7288, Marseille, France; e Pennington Biomedical Research Center, Louisiana State University, Baton Rouge, LA, USA; f Laboratorio de Fisiología Del Ejercicio y Metabolismo (LABFEM), Escuela de Kinesiología, Facultad de Medicina, Universidad Finis Terrae, Chile; g Centre for Muscle Research, Department of Anatomy and Physiology, The University of Melbourne, Melbourne, Victoria, Parkville 3010, Australia; h Developmental and Regenerative Biology Division, Victor Chang Cardiac Research Institute, Darlinghurst, NSW, 2010, Australia; i School of Clinical Medicine, UNSW Sydney, Kensington, NSW 2052, Australia

**Keywords:** Skeletal muscle, FAPs, Adipocytes, Adipogenesis, Obesity, IMAT, Metabolism

## Abstract

Excessive accumulation of intermuscular adipose tissue (IMAT) is a common pathological feature in various metabolic and health conditions and can cause muscle atrophy, reduced function, inflammation, insulin resistance, cardiovascular issues, and unhealthy aging. Although IMAT results from fat accumulation in muscle, the mechanisms underlying its onset, development, cellular components, and functions remain unclear. IMAT levels are influenced by several factors, such as changes in the tissue environment, muscle type and origin, extent and duration of trauma, and persistent activation of fibro-adipogenic progenitors (FAPs). FAPs are a diverse and transcriptionally heterogeneous population of stromal cells essential for tissue maintenance, neuromuscular stability, and tissue regeneration. However, in cases of chronic inflammation and pathological conditions, FAPs expand and differentiate into adipocytes, resulting in the development of abnormal and ectopic IMAT. This review discusses the role of FAPs in adipogenesis and how they remodel IMAT. It highlights evidence supporting FAPs and FAP-derived adipocytes as constituents of IMAT, emphasizing their significance in adipose tissue maintenance and development, as well as their involvement in metabolic disorders, chronic pathologies and diseases. We also investigated the intricate molecular pathways and cell interactions governing FAP behavior, adipogenesis, and IMAT accumulation in chronic diseases and muscle deconditioning. Finally, we hypothesize that impaired cellular metabolic flexibility in dysfunctional muscles impacts FAPs, leading to IMAT. A deeper understanding of the biology of IMAT accumulation and the mechanisms regulating FAP behavior and fate are essential for the development of new therapeutic strategies for several debilitating conditions.

## Graphical Abstract

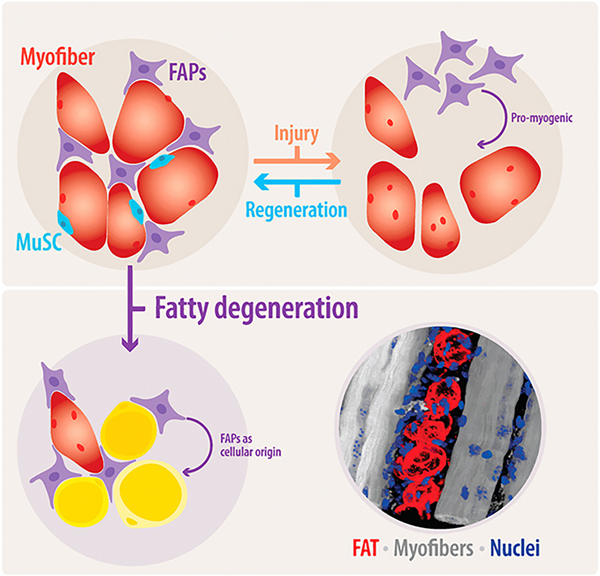

## 1. Introduction

**Fig. 1.**
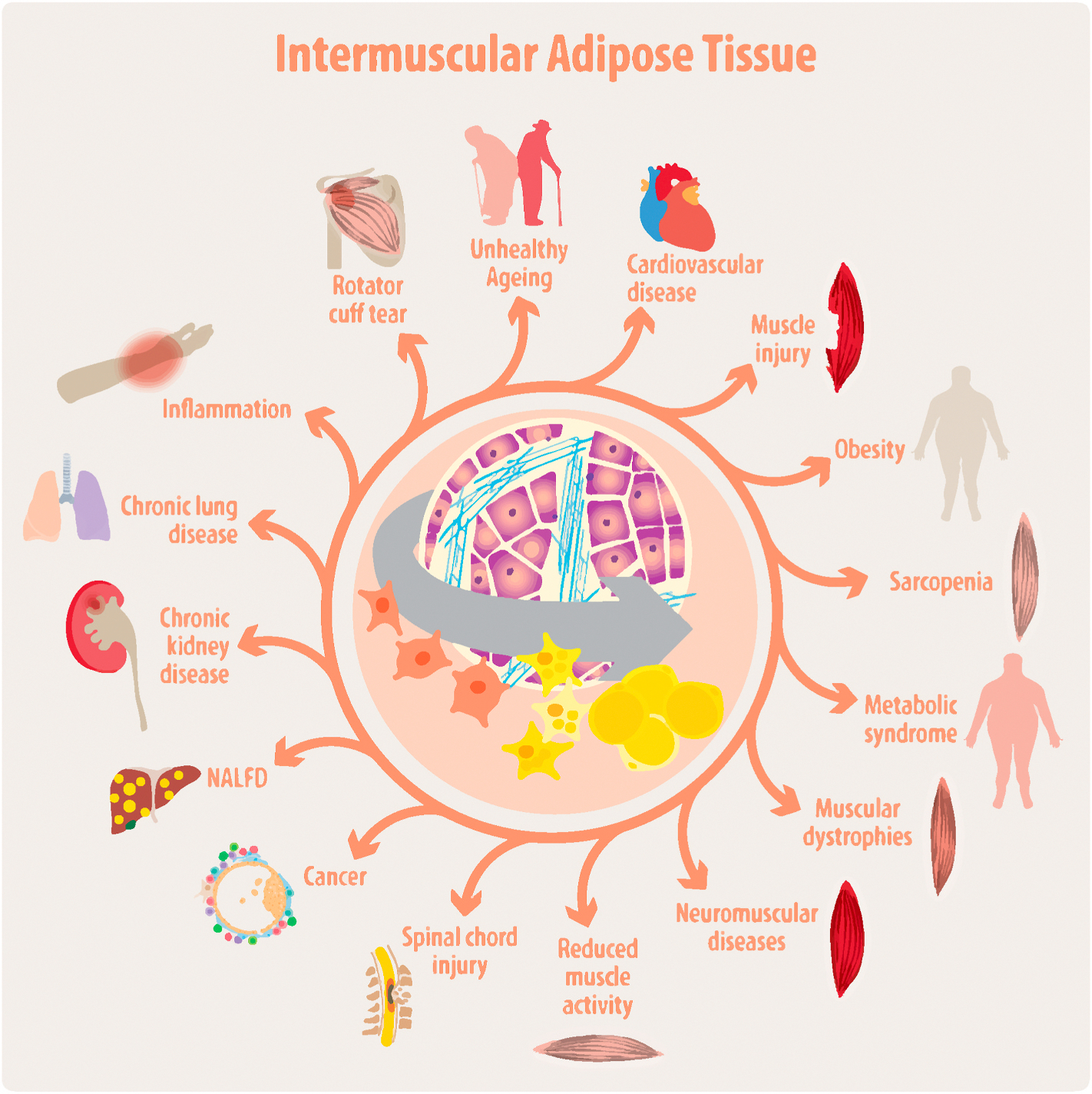
IMAT deposition, a hallmark of chronic fatty infiltration, is exacerbated in a variety of debilitating and chronic human diseases and injuries. This illus - tration highlights the diverse range of pathologies in which IMAT deposition is implicated, including unhealthy aging, obesity, diabetes and metabolic syn - drome, cardiovascular disease, neurode - generative diseases, cancer, and chronic lung and kidney disease.

Normal physical activity and metabolic homeostasis rely on optimal skeletal muscle health and function. Intermuscular adipose tissue (IMAT) is an ectopic fat depot that grows between muscle fibers and surrounding muscle groups. IMAT is a common feature of various chronic diseases in which muscle mass is progressively lost and replaced by adipose and fibrotic tissue. IMAT can be detected in individuals with chronic muscle disease and wasting, inflammatory muscle disorders, sarcopenia (Therkelsen et al. 2013, 2016; Addison et al., 2014), systemic and local immunometabolic and hormonal disturbances, metabolic disorders (i.e., obesity, insulin resistance, type 2 diabetes mellitus [T2DM]) and cardiovascular alterations, including hypertension and heart failure (Manini et al., 2007; Taaffe et al., 2009; Tuttle et al., 2011; Pagano et al., 2018; Burkhart et al., 2019). IMAT infiltration has also been associated with muscle deconditioning due to physical inactivity in the context of unhealthy aging, cancer, chronic kidney and lung disease, and fatty liver disease, where the presence of IMAT increases cardiometabolic mortality and morbidity risks (Fig. 1).

Physical activity is essential for maintaining skeletal muscle mass, oxidative capacity, metabolism, and function. In contrast, reduced physical activity can cause mitochondrial dysfunction and increase the accumulation of lipids in muscle tissue, both within myofibers (such as intramyocellular triglycerides [IMTG]) and in interstitial adipocytes, which are part of the IMAT (Bey and Hamilton 2003; Tarnopolsky et al., 2007; Meex et al., 2010; Leskinen et al., 2013; Hasegawa et al., 2016; Park et al., 2020; Seibert et al., 2020; Tunon-Suarez et al., 2021). Several studies have linked IMAT to impaired muscle functionality following a period of muscle disuse (such as immobilization or detraining) regardless of age, which can lead to disability and reduced quality of life (Manini et al., 2007; Brooks et al., 2008; Popadic Gacesa et al., 2011; Burkhart et al., 2019). Consequently, the economic burden of these conditions on families and healthcare systems can be significant. Despite the negative impacts of IMAT on muscular quality and function, the molecular and cellular mechanisms driving its accumulation are not well understood.

This review provides a comprehensive synthesis of the molecular pathways and cellular interactions regulating IMAT accumulation, with a focus on the role of fibro-adipogenic progenitors (FAPs), the main cell type of origin of IMAT, and their role in maintaining muscle health. We investigated the adipogenic commitment and differentiation of FAPs through consideration of factors such as muscle group-specific location, injury-specific cues, FAP ontogeny, epigenetic and chromatin remodeling, and muscle characteristics. Additionally, we discuss the role of reduced muscle contractile activity in the accretion of lipids into the IMAT and the potential involvement of the regulation of cellular metabolic flexibility within muscle in the expansion of FAPs leading to IMAT accumulation.

Given the high prevalence of chronic conditions, sarcopenia and unhealthy aging, understanding the physiological role of FAPs is crucial for developing effective interventions for muscle tissue maintenance, regeneration and repair. In this context, promoting muscle repair cannot be effective without simultaneously counteracting IMAT development, as these two interventions are expected to act synergistically. Thus, targeting FAPs is a promising therapeutic strategy for mitigating the deleterious effects of chronic conditions and maintaining muscle health.

## 2. Intermuscular adipose tissue in muscle health, regeneration and disease

IMAT is characterized by the accumulation of adipocytes located beneath the outermost muscle fascia within muscle fascicles and between muscle fibers. Low levels of IMAT, in the form of small clusters of perivascular adipocytes, are normal components of muscle in adults without underlying pathology. However, IMAT is often associated with chronic diseases and muscle deconditioning. A severe increase in IMAT and fibrosis, known as fibro-fatty degeneration, is commonly observed in muscle diseases such as Duchenne muscular dystrophy (DMD), inflammatory myopathies, long-term denervation and trauma (Reimers and Finkenstaedt 1997; Chan and Liu 2002; Gladstone et al., 2007; Chang et al., 2018; Feeley et al., 2020).

IMAT accumulation has been linked to reduced muscle loading and physical activity, including prolonged bed rest, limb immobilization, zero-gravity spaceflight (Manini et al., 2007; Pagano et al., 2018; Burkhart et al., 2019), chronic musculoskeletal conditions or muscle trauma (i.e., knee osteoarthritis, low back pain, rotator cuff tears) (Ikemoto-Uezumi et al., 2017; Sions et al., 2017; Trudel et al., 2019), spinal cord injury (Elder et al., 2004; Gorgey and Dudley 2007; Gorgey et al., 2014), sarcopenia (Zoico et al., 2010; Yoshiko et al., 2018), and chronic diseases, including chronic obstructive pulmonary disease (Maddocks et al., 2014), chronic kidney disease (Cheema et al., 2010), alcoholic and non-alcoholic fatty liver disease (Montano-Loza et al., 2016), cancer (Fujiwara et al., 2015; Silva de Paula et al., 2018; Horii et al., 2020), HIV-related wasting syndrome (Torriani et al., 2003), obesity-related disorders, and T2DM (Goodpaster et al. 2000b, 2000c, 2023; Therkelsen et al., 2013). Finally, the accretion of IMAT has also been positively correlated with the progressive deterioration of muscle quality and physical performance in chronic inflammatory myopathies (i.e., inclusion body myositis) and rheumatoid arthritis (Khoja et al. 2018, 2020; Laurent et al., 2022) (Fig. 1, Table 1).

**Fig. 2.**
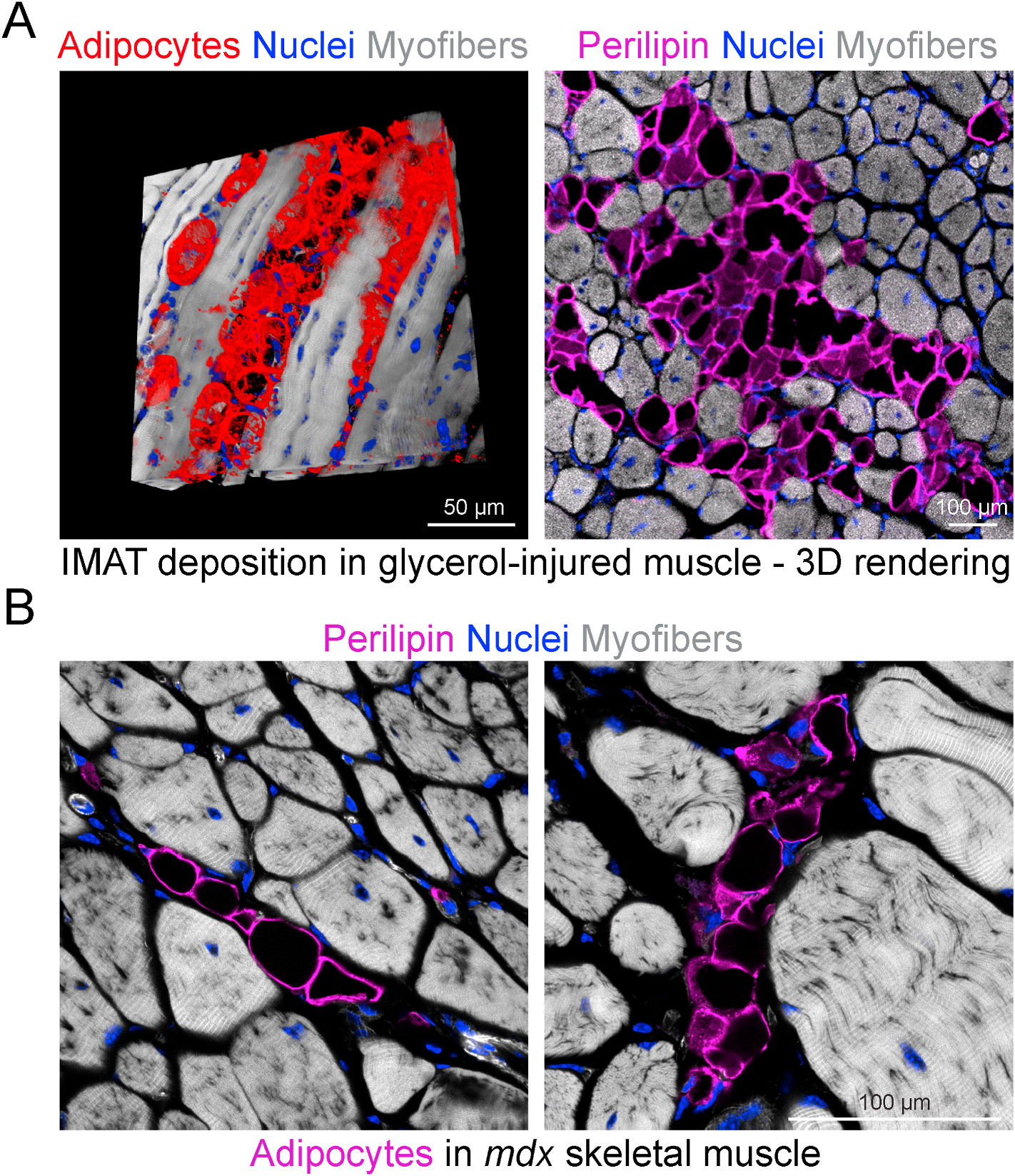
Deposition of IMAT and adipocyte accumulation in mouse models of acute and chronic muscle damage. (A) Three-dimensional rendering illustrating IMAT deposition and enhanced adipocyte accumulation in between regenerating myofibers (left panel), alongside a transverse section imaged via laser confocal microscopy displaying accumulated Perilipin^+^ adipocytes following glycerol-induced damage (right panel). (B) Confocal microscopy images demonstrating the abundance of Perilipin adipocytes within the muscle stroma of dystrophic *mdx* limb skeletal muscles.

In animal models, extensive IMAT formation occurs after a single round of glycerol (GLY) muscle injury (Kawai et al., 1990; Pisani et al., 2010a; Lukjanenko et al., 2013; Mahdy et al., 2015; Pagano et al. 2015, 2019; Biltz and Meyer 2017; Biltz et al., 2020; Xu et al., 2021) (Fig. 2A). Recently, we demonstrated that the accumulation of adipocytes after GLY-induced injury to the tibialis anterior muscle was five times greater than that caused by gold-standard cardiotoxin (CTX) injury in CD1 wild-type mice (Waisman et al., 2021). Tracking with increased IMAT, we also found that myofiber regeneration in the tibialis anterior was reduced with GLY-induced injury compared to CTX injury (Waisman et al., 2021). Notably, because IMAT development following GLY-induced injury tends to persist beyond the expected completion of the regeneration process in 21 days, it shares some of the characteristics of a chronic model. Taken together, these data highlight the potential of the GLY injury model as an effective tool for inducing chronic IMAT infiltration and thus investigating the underlying mechanisms of muscle disease and aging. In summary, IMAT development seems to be a hallmark of acute and chronic conditions, suggesting that all these conditions interfere with FAP homeostasis.

## 3. Fibro-adipogenic progenitors as the main progenitor cells of adipocytes in IMAT

### 3.1. Identification and main characteristics of FAPs

FAPs are a diverse group of stromal non-myogenic cells that play crucial roles in maintaining and repairing muscle tissues. FAP identification was assessed by the expression of mesenchymal cell surface markers such as Sca-1, CD34, and platelet-derived growth factor receptor alpha (PDGFRα). The latter is considered the gold-standard marker for identifying FAPs (Joe et al., 2010; Pisani et al., 2010a; Uezumi et al., 2010; Uezumi et al., 2011). FAPs can differentiate into fibroblasts and adipocytes both *in vitro* and *in vivo* and are responsible for depositing fibrotic and adipose tissues in response to muscle injury (Joe et al., 2010; Uezumi et al. 2010, 2014).

Later studies further expanded our understanding and revealed several important characteristics of FAPs. First, FAPs were identified as the primary cell type source of fibrosis in a widely used model of DMD, the *mdx* mouse (Uezumi et al., 2011). Second, despite being precursors derived from a Pax3-nonmyogenic lineage (Pisani et al., 2010b; Liu et al., 2012), the ablation of FAPs significantly impaired the regeneration of acutely injured muscles, which demonstrated a crucial role in muscle regeneration and homeostasis (Pisani et al., 2010b; Roberts et al., 2013; Uezumi et al. 2014, 2021; Wosczyna et al., 2019). Third, adipogenic FAPs exhibit a cell surface marker profile similar to that previously characterized for progenitor cells derived from white and brown adipose tissues (Rodeheffer et al., 2008; Uezumi et al., 2010; Pannérec et al., 2013; Arrighi et al., 2015). A second set of studies investigated the regulatory mechanisms controlling FAP activity and behavior. FAPs are highly responsive to the type 2 innate cytokine IL-4/IL-13 released by eosinophils (Heredia et al., 2013), and FAP survival is tightly controlled by cross-talk with inflammatory macrophages, which involves induction of apoptosis via tumor necrosis factor-alpha (TNF-α) and release of the mitogenic cytokine transforming growth factor-beta 1 (TGF-β1) (Lemos et al., 2015). Additionally, FAP fate depends on balanced cross-talk between pro-mitogenic and pro-fibrotic signals, TGF-β1 and platelet-derived growth factors (Contreras et al. 2019b, 2020). Other mechanisms regulating FAP behavior include ciliation dynamics and activation of hedgehog intercellular signaling (Kopinke et al., 2017); cell-to-cell contact signals, such as the neurogenic locus notch homolog protein (NOTCH) (Marinkovic et al., 2019); the juxtacrine effect of factors released by damaged myofibers (Hogarth et al., 2019); the regulation of the adipogenic master regulator peroxisome proliferator-activated receptor-gamma (PPARγ) transcriptional activity (Reggio et al., 2019); the autocrine and paracrine effects of Wingless-type MMTV integration site (WNT) ligands; the subsequent activation of the WNT/β-catenin signaling cascade and metabolic cues (Reggio et al., 2020a); and microheterogeneity regulation of FAP differentiation and cell states (Giuliani et al., 2021).

In parallel, several studies have demonstrated that FAPs are necessary for the activation and differentiation of myogenic progenitors. This critical role is exerted through the secretion of pro-myogenic paracrine and juxtacrine factors (Joe et al., 2010; Murphy et al., 2011; Mozzetta et al., 2013; Roberts et al., 2013; Lukjanenko et al., 2019; Uezumi et al., 2021). *In vitro* co-culture and conditioned media from activated FAPs have been shown to increase the myoblast fusion rate and myotube diameter (Mathew et al., 2011; Mozzetta et al., 2013; Madaro et al., 2018). Moreover, ablation of FAPs has been associated with muscle atrophy, muscle weakness, a reduction in muscle stem cells (MuSCs), a fast-to-slow shift in fiber type, neuromuscular junctions and Schwann cell degeneration (Murphy et al., 2011; Liu et al., 2012; Roberts et al., 2013; Wosczyna et al., 2019; Uezumi et al., 2021). Overall, research on FAPs is still in its infancy, and further studies are needed to fully understand their complex biology.

### 3.2. Differentiation routes and fates of FAPs

FAPs represent a population of multipotent progenitor cells in the muscle interstitium, and a significant proportion reside in the vicinity of blood vessels, where they act as perivascular cells rather than pericytes (Joe et al., 2010; Uezumi et al., 2010; Santini et al., 2020; Sono et al., 2020; Theret et al., 2021). Although their main differentiation routes guide them toward fibrogenic and adipogenic lineages, they can acquire other cell fates under specific stimuli. Potentially, a small subset of injury-activated FAPs may differentiate into endothelial cells in response to hypoxia and glucose deprivation (Ollitrault et al., 2021). In severe inflammatory and chronic disease conditions, muscle FAPs acquire an osteogenic phenotype, as observed in mouse models of heterotopic ossification and severe muscle disease (Lees-Shepard et al., 2018; Eisner et al., 2020; Mázala et al., 2020; Julien et al., 2021). Despite their multipotent differentiation capacity, FAPs traditionally were not thought to contribute to the myogenic lineage under normal conditions. This fate limitation has been related to the expression and activity of the nuclear envelope protein Prdm16 in FAPs (Biferali et al., 2021). This transcription factor is typically expressed in Myf5^+^/Pax7^+^ myogenic and brown-fat progenitors and acts as a fate switch regulating the choice of these cells to become either muscle or brown fat cells (Seale et al., 2008; An et al., 2017). Moreover, knockdown of *Prdm16* in brown fat precursors induced myogenesis, whereas its overexpression promoted brown adipocyte differentiation in myogenic cells. In FAPs, Prdm16 inhibits the expression of a myogenic-like gene profile, an activity exerted in conjunction with the histone methyltransferases G9a and GLP, to confine myogenic genomic loci within a silent heterochromatin-like compartment underneath the nuclear lamina (Biferali et al., 2021). In line with these findings, the selective inhibition of G9a induced an anti-adipogenic effect on FAPs and promoted myotube formation (Randazzo et al., 2022). These results suggested that Prdm16 and G9a-mediated H3K9 histone methylation switch off myogenic differentiation in FAPs, thus confirming their stable non-myogenic fate. While most FAPs are generally not involved in muscle cell formation, recent research suggests that certain types of FAPs may contribute to creating muscle cells. Specifically, a study in 2023 by Flynn and colleagues identified a group of that express the gene Homeobox A11 (*Hoxa11*). These cells have been shown to help form new myogenic cells that then become part of the muscle fibers (Flynn et al., 2023). This finding supports the emerging idea that some interstitial progenitor cells, rather than the traditional Pax7-expressing MuSCs, serve as a significant source for adult muscle tissue maintenance *in vivo* (Flynn et al., 2023).

Regarding FAP adipogenic capacity, comparative *in vitro* and *ex vivo* studies have shown that FAPs share similar adipogenicity and functional properties with adipocyte precursors (APCs) from subcutaneous and visceral fat depots (Arrighi et al., 2015; Laurens et al., 2016; Sachs et al., 2019). Arrighi et al. (2015) demonstrated that adipogenesis, expression of adipogenic markers, triglyceride synthesis and degradation were comparable between muscle FAP-derived adipocytes and APC-derived adipocytes. However, insulin failed to stimulate glucose uptake or insulin signaling in FAPs. Hence, the authors concluded that the IMAT behaves as a fat compartment that is insensitive to insulin (Arrighi et al., 2015). Notably, the differentiation media used in the study contained 860 nM insulin (0.5 μg/ml), which is 43-fold greater than that of standard adipogenic cocktails (20 nM or 11.6 ng/ml). This high concentration of insulin may have yielded FAP-derived adipocytes unresponsive to its effect since a previous study revealed that prolonged insulin treatment dose-dependently induced insulin resistance and impaired mitochondrial function in contractile cells of lymphatic vessels (Lee et al., 2017a). Moreover, the idea of FAPs being insensitive to insulin was further refuted by transcriptomic analyses revealing the expression of the insulin receptor and downstream insulin effectors, such as the serine-threonine protein kinase AKT and the glucose transporter GLUT4 (SLC2A4), in isolated human IMAT explants (Sachs et al., 2019). In fact, the expression of these markers correlated with the insulin sensitivity of the donor and was downregulated in patients with low insulin sensitivity (Sachs et al., 2019).

Additionally, FAPs have been shown to differentiate into brown-like adipocytes expressing uncoupling protein 1 (UCP-1) in response to β-adrenergic stimulation or cold exposure in both mice and humans (Uezumi et al., 2014; Gorski et al., 2018; Lee et al. 2020b, 2020c). However, not all studies have evaluated this ability (Pisani et al., 2010b; Wosczyna et al., 2012; Arrighi et al., 2015), and the results appear dependent on cell culture conditions and differentiation protocols (Gorski et al., 2018). Nonetheless, the specific conditions favoring white or brown/beige fat cell differentiation in FAPs *in vivo,* have yet to be determined. In a translational setting, transplantation of UCP-1^+^ FAPs, described as beige-FAPs, seems to improve muscle function and reduce fatty degeneration in a mouse model of rotator cuff tear (Lee et al., 2020c).

Despite the well-established roles of FAPs in muscle maintenance and regeneration, excessive activation and subsequent differentiation into adipocytes lead to pathological formation of IMAT and fibro-fatty degeneration in muscle diseases (Uezumi et al., 2011; Paylor et al., 2014; Lemos et al., 2015; Contreras et al. 2016, 2019c; Ikemoto-Uezumi et al., 2017; Hogarth et al., 2019; Reggio et al. 2019, 2020b; Farup et al., 2021; Liu et al., 2021; Shirasawa et al., 2021; Vumbaca et al., 2021; Davies et al., 2022). FAPs also contribute to IMAT formation in response to chronic systemic diseases and muscle injury (Fig. 1), and this adipogenic potential is influenced by the interaction between FAPs, surrounding cells, and the prevailing cellular environment, as demonstrated in transplantation studies (Joe et al., 2010; Uezumi et al., 2010; Liu et al., 2012; Uezumi et al., 2014; Kopinke, Roberson et al. 2017; Stumm et al., 2018; Hogarth et al., 2019; Contreras and Harvey 2023).

Nonetheless, immune cells may also contribute to ectopic IMAT adipogenesis, perhaps to a small degree. For instance, single-cell RNA-seq analysis revealed a subpopulation of muscle-resident myeloid-derived cells that expressed adipocyte-enriched genes and accumulated lipids upon adipogenic induction *in vitro* (Xu et al., 2021). Future studies that include parabiosis and detailed lineage tracing should help to answer these questions.

### 3.3. Cellular and functional heterogeneity of muscle FAPs

Muscle FAPs are not homogeneous but rather constitute a group of cells with varying characteristics and roles *in vivo* (Collins and Kardon 2021; Contreras et al., 2021b). The diversity of FAPs relies on the anatomical location of the muscle in which they reside (Muhl et al., 2020; Contreras et al., 2021b) and dynamic changes in the microenvironment after muscle injury and in diseased states (Malecova et al., 2018; Marinkovic et al., 2019; Zhang et al., 2019; Contreras et al., 2020; Rubenstein et al., 2020; Giuliani et al., 2021). The FAP secretome and its functions are highly dynamic and ensure that the regenerative microenvironment sustains muscle health and integrity. This section explores the factors that influence the emergence of diverse FAP cellular states and fates and how this adaptive response fosters a favorable pro-regenerative microenvironment that supports muscle regeneration or may lead to debilitating IMAT formation in muscle diseases. Additionally, we describe the transcriptional profiles and functional characteristics of distinct FAP states from embryonic FAPs to quiescent FAPs in adult muscles and their activation to a promyogenic state after injury and the presence of other FAP subtypes under normal and pathological conditions and in diseases.

#### 3.3.1. Heterogeneity of tissue of origin of FAPs

During embryonic development, muscle connective tissue progenitor cells, including FAPs, originate from different embryonic structures (reviewed previously (Helmbacher and Stricker 2020)). Three major embryonic origins have been identified for muscle-resident FAPs: 1) the neural crest for craniofacial and neck muscles, and most of the cartilage, bone, dentine and other connective tissues of the head (Diogo et al., 2015); 2) the lateral plate mesoderm for trunk and limb muscles; and 3) the pleuroperitoneal folds for the diaphragm (Contreras et al., 2021b). Cells emerging from the cranial neural crest participate in the formation and shape of muscles on the shoulder girdle, neck, and face and are the source of FAPs in these regions. Subsequently, these FAPs may eventually form IMATs in rotator cuff tears and craniofacial muscle fibro-fatty infiltrating disorders (Paylor et al., 2014; Lee et al., 2020a). Lateral plate mesoderm and somites form axial trunk muscles and muscles of the limbs and are the source of FAPs, contributing to the formation of IMAT within these muscle groups (Lemos et al., 2012; Paylor et al., 2014). Finally, pleuroperitoneal folds are transient non-myogenic structures that develop in conjunction with the diaphragm and give rise to the central tendon and FAPs in mice (Merrell et al., 2015). They are an important source of myogenic promoting signals during diaphragm development. In contrast, FAPs contribute to IMAT in the diaphragm in adult mice in response to a high-fat diet (HFD) (Buras et al., 2019).

A population of embryonic FAPs expressing the odd-skipped related transcription factor 1 (Osr1) zinc-finger transcription factor was identified (Vallecillo-García et al., 2017). These Osr1^+^ FAPs exhibited a transcriptional program associated with extracellular matrix (ECM) biogenesis and matrisome assembly; this program included collagen chains (*Col6a1*, *Col6a2*, and *Col6a3*), *lumican*, *matrillin*, *decorin*, *fibromodulin*, and *nidogen 2*. Osr1^+^ FAPs express the chemokine *Cxcl12* and the growth factor bone morphogenetic protein 4 (Bmp4), both of which are known to promote myogenesis (Vallecillo-García et al., 2017). Thus, Osr1^+^ FAPs create a favorable environment supporting the proliferation and survival of myogenic progenitors during development by expressing ECM components and promyogenic factors (Vallecillo-García et al., 2017). In a recent study, scRNA-seq revealed six clusters of embryonic PDGFRα^+^ cells in developing muscle, from which cluster 4 showed high *Osr1* expression and other genes associated with stemness and developmental processes (Leinroth et al., 2022). The other five clusters followed three distinctive differentiation trajectories. First, an immune responsive population with high fibrogenic and adipogenic potency. Second, highly osteogenic cells expressing *clusterin* and *hemicentin-1*. Finally, neuromuscular junction (NMJ)-associated cells with no adipogenic potential (Leinroth et al., 2022). Additionally, although the number of embryonic Osr1^+^ FAPs declines in the muscle interstitium soon after birth, their progeny contributes to FAP populations in adult muscle, giving rise to Sca1^+^ and PDGFRα^+^ FAPs (Vallecillo-García et al., 2017; Stumm et al., 2018).

Regarding their location, adult FAPs from different muscle locations differ in their number, functional proliferation, and adipogenic properties. Lee et al. (2020d) demonstrated that the masseter, rotator cuff, and paraspinal muscles have a greater proportion of FAPs per gram of tissue than does the gastrocnemius and tibialis anterior muscles of the hindlimb in mice (Lee et al., 2020a). Furthermore, FAPs isolated from these muscles, especially from the shoulder muscles, exhibit differential proliferative activity and adipogenic potency (Lee et al., 2020a), which is relevant considering the rapid onset of IMAT after rotator cuff tears. However, whether the differences in the functional capabilities of FAPs are related to their heterogeneous embryonic origin or whether changes occur later in their developmental history has not been studied. Apart from FAP ontogeny, this functional variability could also be attributed to variable pro-adipogenic patterns across muscle groups, *in situ* injury-specific cues, intrinsic epigenetic mechanisms, and differences in muscle fiber type composition, innervation, vascularity, and biomechanical function. Given the complexity of the adipogenic fate of muscle FAPs, we propose that all these variables could determine the fate of FAPs and, therefore, need to be considered when designing interventions to enhance muscle tissue regeneration and repair. Regardless of their embryonic origin and location, FAPs are the source of signals necessary to promote myogenic growth during development and regeneration, metabolic alterations, and systemic nutritional cues (Paylor et al., 2014; Mogi et al., 2016; Buras et al., 2019), which demonstrates functional convergence in supporting muscle homeostasis.

### 3.4. The potential pro-regenerative role of FAP-derived adipocytes in homeostatic muscle

Although IMAT accumulation is observed in various pathological conditions, even healthy humans and rodents have small amounts of IMAT in most muscles. However, the physiological role of IMAT has not been determined. Small numbers of adipocytes can be observed in adult muscles, suggesting they may play a supportive and homeostatic role in muscle (Pagano et al., 2018; Sachs et al., 2019; Jakobsen et al., 2021). An example of the potential homeostatic role of FAP-derived adipocytes is illustrated by the perivascular adipocytes found in the myotendinous junction and lining the venous arcade in the diaphragm muscle of mice (Stuelsatz et al., 2012; Sono et al., 2020)). These observations were recently corroborated in different muscle groups of healthy humans and mice (Zhang and Wang 2015; Jakobsen et al., 2021), suggesting an unappreciated role in regulating muscle-tendon transitional zones and myotendinous junctions. In muscle regeneration, genetic elimination of committed/differentiating *Ap2*/*Fabp4*^+^ muscle adipocytes results in impaired regeneration (Liu et al., 2012; Dammone et al., 2018). Mice lacking *Pparg* exhibit no ectopic IMAT formation during muscle regeneration. This absence of IMAT disrupted muscle regeneration and injury-induced MuSC expansion and myogenesis, potentially through alterations in the inflammatory response (Dammone et al., 2018). However, further research is needed to determine the role of FAP-derived adipocytes and their potential contributions to these physiological processes, as well as potential therapeutic applications in muscle maintenance, regeneration and repair.

### 3.5. Challenges to the ectopic IMAT dogma: subcutaneous adipose stromal cells as a novel source of IMAT

Muscle-resident FAPs are considered to be the main cell type of origin for ectopic adipogenesis, yet Sengenès’ group challenged this idea by showing that an 8-week HFD can mobilize CXCR4-expressing subcutaneous adipose stromal cells into skeletal muscle (Girousse et al., 2019). This involved the CXCL12/CXCR4 axis, where high CXCL12 levels facilitate retention of adipose stromal cells in subcutaneous adipose tissue (SCAT), whereas low CXCL12 levels favor egress from SCAT. To visualize the trafficking and relocation of these cells in response to a HFD, a piece of subcutaneous adipose tissue from CD34^EGFP^ and Ad-Cre/Zs1Green mice was grafted into the SCAT of wild-type mice that were subsequently fed a HFD for 8 weeks (Girousse et al., 2019). First, CD34^−^/CD45^−^/CD31^−^/EGFP^+^/SCA1^+^ progenitor cells relocated from the SCAT graft into the quadriceps muscle, together with Zs1Green-labeled adipocytes found in the same muscle group. Remarkably, they found that promoting adipose stromal cell egress from SCAT with the CXCR4 antagonist AMD3100 was sufficient to increase IMAT deposition and to impair glucose tolerance to levels comparable to those of HFD (Girousse et al., 2019). These findings suggest that blocking the mobilization of these stromal cells might prevent ectopic IMAT deposition and metabolic disturbances. However, the function of AMD3100 in muscle-resident FAPs and MuSCs was not further evaluated.

Recently, the same group showed that SCAT-derived stromal cells serve, not only as a source of muscle FAPs in HFD-fed mice, but also after muscle injury. In this situation, SCAT-derived FAPs promoted muscle regeneration in a manner similar to FAPs (Sastourné-Arrey et al., 2023). The authors found that the number of FAPs significantly increased 24 h after CTX or GLY-induced injury in the quadriceps muscle (Sastourné-Arrey et al., 2023). CD34^EGFP^ grafting experiments suggested that the increase in FAP content occurred via injury-induced stromal cell migration from SCAT into muscle and not solely by proliferation of muscle-resident FAPs. They proposed a model in which the podoplanin-mediated interaction between adipose stromal cells and platelets influences the infiltration of SCAT-derived stromal cells into damaged muscles (Sastourné-Arrey et al., 2023). Blocking stromal cell egression by depleting platelets resulted in impaired expression of late myogenic markers and muscle regeneration, suggesting SCAT-derived stromal cells play an early pro-regenerative and supportive role in the damaged muscle microenvironment, as is known for muscle-resident FAPs (Sastourné-Arrey et al., 2023). These findings suggest that SCAT interacts with skeletal muscle through the mobilization of adipogenic progenitors. However, identifying muscle-resident or adipose tissue-exported FAPs using lineage tracing is challenging because these cells share cell-specific markers, such as CD34. Moreover, CD34 is expressed in MuSCs, circulatory cells, and the endothelium (Dooley et al., 2004; Rodeheffer et al., 2008; Pisani et al., 2010b; Mitterberger et al., 2012).

#### 3.5.1. In search of true adipogenic progenitor cells: a dual fate

Single-cell and single-nucleus transcriptomics together with mass cytometry advances have enabled an unprecedented description of the transcriptional heterogeneity and protein dynamic transitions of various FAP subpopulations and cell states. For instance, Petrilli et al. (2020) provided a compendium of mass cytometry FAP markers in mice and humans, validating previously described markers. Additional studies have expanded this information and detailed how FAP subpopulations change their transcriptional signatures and secretome depending on their activation and progression during the regenerative process and in response to a HFD or exercise (Malecova et al., 2018; Scott et al., 2019; Muhl et al., 2020; Oprescu et al., 2020; Yang et al., 2021; Fitzgerald et al., 2023). In this section, we summarize the main identity markers and functional properties of different FAP subtypes and states during adult muscle regeneration, repair and aging.

##### 3.5.1.1. Quiescent FAPs.

In unperturbed adult muscle, quiescent FAPs are identifiable based on their lack of protein markers of endothelial cells (CD31^−^), hematopoietic cells (CD45^−^), myogenic cells (SM/C-2.6^−^, CD56^−^, Pax7^−^and α7-integrin^−^), and erythroid cells (Ter119^−^). Most importantly, FAPs are characterized by high expression levels of mesenchymal stromal cell surface markers, including PDGFRα, SCA1, CD34, the angiopoietin receptor Tie2, and THY-1 (known as CD90), and the transcription factors Osr1 and TCF7L2. Malecova et al. (2018) were the first to utilize single-cell approaches to identify distinct subpopulations of SCA1^+^CD34^+^ murine FAPs. These subpopulations were characterized by the expression of the vascular cell adhesion molecule Vcam1 and the angiopoietin-1 receptor Tie2 (Malecova et al., 2018). In healthy adult muscle, most quiescent FAPs expressed low levels of *Tie2* (Tie2^low^). Next, based on the differential expression of SCA1 and CD34 at the single-cell level, a follow-up multiparametric mass cytometry study identified distinct subpopulations of muscle FAPs from juvenile (6-week-old) WT mice, CTX-injured muscles, and muscles from *mdx* mice (Marinkovic et al., 2019), where quiescent FAPs expressed low levels of both antigens (SCA1^low^CD34^low^). In parallel, we distinguished the activation and differentiation states of pro-fibrotic FAPs based on the expression of PDGFRα and TCF7L2, with both markers being highly expressed in a quiescent state (PDGFRα^high^TCF7L2^high^ FAPs) (Contreras et al. 2019b, 2020). Upon trauma, mouse and human TCF7L2^+^ FAPs are located preferentially in areas surrounding regenerative muscle fibers (Contreras et al., 2016; Mackey et al., 2017). Although many of the aforementioned markers are enriched in FAPs, they are not exclusive to these cells, and their expression level varies along non-homeostatic conditions (reviewed by (Contreras et al., 2021b)). Another two distinct FAP subpopulations were identified in humans using scRNAseq based on the expression of *FIBRILLIN-1* (FBN1^+^) and *LUMICAN* (LUM^+^) in unperturbed muscle obtained from donors aged 15–75 years (Rubenstein et al., 2020). These subsets differed in their location within muscle and expressed different collagen genes. Whereas FBN1^+^ FAPs are located preferentially in the perimysium, LUM^+^ FAPs are located closer to the endomysium (Rubenstein et al., 2020). Finally, the transcription factor hypermethylated in cancer 1 protein (HIC1) is expressed in quiescent FAPs in muscles and the heart (Scott et al., 2019; Soliman et al., 2020; Contreras et al., 2021b). *Hic1* deletion experiments suggest that HIC1 downregulation is required for the activation of cardiac FAPs post myocardial infarction (Soliman et al., 2020).

##### 3.5.1.2. Injury-induced activation of FAPs.

In adult muscles, FAP cell dynamics involve the activation of a quiescent pool of FAPs in response to various myotrauma-related autocrine, paracrine and juxtacrine factors, especially those linked to the rapid acute inflammatory response. In response to acute muscle damage, the composition of the interstitial milieu changes, allowing previously quiescent FAPs to be rapidly activated, enter the cell cycle and proliferate, resulting in a significant increase in their number. This proliferative wave peaks between day 3 and day 5 after injury (Joe et al., 2010; Lemos et al., 2015; Contreras et al. 2019c, 2020) (reviewed by (Theret et al., 2021)). Notably, gene expression analysis revealed that the majority of muscle cycling FAPs exhibit upregulated expression of genes associated with cell cycle progression as early as day 2 following acute injury, including *Mki67*, *Top2a*, *Stmn1*, and *Birc5* (Contreras and Harvey 2023). These findings further support the findings of previous studies using mass spectrometry-proteomic profiling, which revealed increased expression of proteins involved in the cell cycle and DNA replication in FAPs in response to acute muscle damage and muscular dystrophy (Marinkovic et al., 2019; Reggio et al., 2020a).

The expression of mesenchymal cell markers, such as PDGFRα and TCF7L2, which is characteristically high in quiescent cells, is downregulated during FAP activation in response to tissue damage (Contreras et al. 2019b, 2020). In contrast, the number of SCA1^+^CD34^+^ FAPs and Osr1^+^ FAPs is increased (Vallecillo-García et al., 2017; Stumm et al., 2018; Marinkovic et al., 2019). Together, these transcriptional and proteomic changes indicate the activation of quiescent FAPs. Upon injury, FAPs are also recognized by gene expression patterns that are essential for adult myogenesis, ECM remodeling, and immune cell attraction, leading to efficient muscle regeneration (Scott et al., 2019; De Micheli et al., 2020a; Oprescu et al., 2020; Contreras and Harvey 2023).

Although predominant during embryonic development, Osr1^+^ FAPs are rare in mature muscles, but they rapidly increase in number following acute muscle injury and undergo apoptosis shortly after this stage. Moreover, the decrease in survival of injury-activated Osr1^+^ FAPs can give rise to a portion of TCF7L2^+^ FAPs and interstitial adipocytes after injury (Stumm et al., 2018). These findings suggest that adult Osr1^+^ FAPs can reactivate a developmental program that supports muscle tissue regeneration (Vallecillo-García et al., 2017; Stumm et al., 2018). The pro-regenerative activity of Osr1^+^ FAPs was confirmed to be functionally linked to *Osr1* gene function, as conditional deletion of *Osr1* in FAPs drives excessive FAP fibrogenesis, favoring muscle fibrosis and impairing muscle regeneration (Kotsaris et al., 2023).

##### 3.5.1.3. Role of FAPs in early post-injury stage.

During the very early stages of muscle regeneration, activated FAPs dynamically express immunoreactive factors such as CC motif chemokine ligands (Ccl2, Ccl5, and Ccl11), C-X-C motif ligands (Cxcl3, Cxcl5, and Cxcl14), CCN2/CTGF, and Timp1, among other factors necessary for the recruitment, expansion, and maturation of immune cells (Lukjanenko et al., 2019; Scott et al., 2019; De Micheli et al., 2020a; Oprescu et al., 2020; Rubenstein et al., 2020; Contreras et al., 2021b; Contreras and Harvey 2023). This immune-like signature suggests that activated FAPs act as immunomodulatory cells that engage in active cross-talk with each other and with other muscle-resident and non-resident cells to regenerate muscle and restore function (Contreras et al., 2021b; Theret et al., 2021). Following this immune-regulatory phase, FAPs produce several trophic and myogenic factors that generate a proper cellular milieu for muscle repair and maintenance. First, FAPs produce collagen VI, which helps to maintain MuSC quiescence and self-renewal during muscle regeneration (Urciuolo et al., 2013). Second, in mature muscles, the pro-myogenic role of FAPs favors a pro-regenerative MuSC behavior and fate via the secretion of factors such as insulin-like growth factor-1 (IGF-1), interleukin-6 (IL-6), WNT ligands, follistatin, and bone morphogenetic protein-3b (Bmp3b, or growth differentiation factor 10, Gdf10) (Joe et al., 2010; Mozzetta et al., 2013; Roberts et al., 2013; Farup et al., 2015; Scott et al., 2019; Oprescu et al., 2020; Reggio et al., 2020b; Rubenstein et al., 2020; Uezumi et al., 2021; Norris et al., 2023). Activated FAPs and their progeny actively secrete a plethora of matricellular factors (e.g., CCN2/CTGF, WNT1 inducible signaling pathway protein 1 [WISP1]) and matrisome components, including laminins, collagens, fibronectin, lumican and decorin, among others (Contreras et al., 2016; Mackey et al., 2017; Scott et al., 2019). The secretion of FAP-derived WISP1 is required to induce MuSC proliferation, commitment, and myogenic differentiation (Lukjanenko et al., 2019). This promotes MuSC activation and differentiation, myocyte fusion, and the formation and maintenance of NMJ (Mackey et al., 2017; Uezumi et al., 2021). These are critical steps for maintaining a regenerative and pro-myogenic niche (Heredia et al., 2013; Contreras et al. 2019c, 2020; Scott et al., 2019; Mázala et al., 2020).

##### 3.5.1.4. Role of FAPs in advanced post-injury stage.

Three different subsets of FAPs have been described to exist at an advanced postinjury timepoint. First, a subgroup of Tie2^high^ FAPs, which exhibit a gene signature critical for muscle regeneration, including genes involved in the chemotaxis of blood cells, maturation of dendritic cells, and neoangiogenesis, was found (Malecova et al., 2018; Endo et al., 2023). Second, a population of FAPs expressing Delta-like Noncanonical NOTCH Ligand 1 (Dlk1, also known as preadipocyte factor-1; Pref-1) appears. This subset of genes exhibited a signature characterized by the expression of genes associated with the complex imprinting pattern, including *B830012L14*, *Rik*, *Meg3*, *Airn*, *Peg3* [Pw1], *Zim1*, *H19*, and *Igf2*. Whether imprinting plays a role in a specific functional transition has not been studied. Notably, Dlk1/Pref-1 has been identified as a marker of adipose precursors, and ablation of Pref-1-expressing cells was shown to prevent adipose tissue development and expansion, thus demonstrating the requirement of Pref-1^+^ cells for adipogenesis (Hudak et al., 2014). However, whether the Dlk1^+^FAP population represents a pre-adipocyte state during muscle regeneration has yet to be determined. Third, a small fraction of Osr1^+^ FAPs has been described (Stumm et al., 2018; Oprescu et al., 2020). These cells are characterized by the expression of cell signaling-related genes, such as *Ccl1*, *Bmp4*, *Bmp5*, and the anti-adipogenic factor *Wnt5a* (Oprescu et al., 2020). Interestingly, based on the anti-adipogenic effect of recombinant WNT5A and *in vitro* treatment with an inhibitor of the upstream β-catenin kinase glycogen synthase kinase (GSK), Reggio et al. (2020a) suggested that WNT5A serves as an autocrine adipogenic break and that FAPs secrete WNT5A to restrict the development of IMAT at a later time point during regeneration (Reggio et al., 2020b). At the end of regeneration, some Osr1^+^ FAPs diverged into two populations, identified as the dipeptidyl peptidase 4 (Dpp4)^+^ FAP and Cxcl14^+^ FAP subpopulations (Oprescu et al., 2020). Previously, Dpp4-expressing cells were identified as bona fide adipogenic progenitor cells located in the reticular interstitium of white adipose tissue (Merrick et al., 2019). The adipogenic potential of Dpp4^+^ and Cxcl14^+^ FAP subpopulations in skeletal muscle remains to be determined, though we speculate that Dpp4^+^ FAPs may exhibit greater adipogenic properties compared to Cxcl14^+^ FAPs. Additionally, beyond their differentiation capabilities, Dpp4^+^ FAPs appear to possess neuroprotective effects, playing a critical role in the maturation and maintenance of the neuromuscular system (Kim et al., 2022). This neuroprotective function is mediated through the expression of Survival of Motor Neuron protein (SMN), which is essential for the proper function and survival of motor neurons. Through the expression of the SMN-stabilizing deubiquitinase Bap1, Dpp4^±^ FAPs influence NMJ maturation and motor neuron homeostasis (Kim et al., 2022).

##### 3.5.1.5. Characteristics of pathology-associated FAP subtypes.

In pathological settings, FAP states, subtypes, and selective evolution are usually modulated by the microenvironment. In contrast to the critical transition to a pro-myogenic supporting role in muscle regeneration, the persistence of activated and proliferative FAPs beyond a critical period during muscle repair allows the survival of (an) inducible subpopulation (s) of FAPs capable of responding to prevailing inflammatory, pro-fibrotic and/or pro-adipogenic signals, which should define their functional capabilities that may ultimately compromise muscle regeneration and metabolic homeostasis through the formation of fibrosis and IMAT. Malecova et al. (2018) first described a pro-fibrotic-like Vcam1^+^ FAP subgroup appearing in association with a widespread inflammatory response to acute muscle damage and in dystrophic muscles from 2- to 3-month-old wild-type and *mdx* mice (Malecova et al., 2018). Additionally, FAPs from dystrophic mice exhibited a robust adipogenic phenotype *in vivo* and *in vitro*, a phenotype linked to the loss of NOTCH-mediated repression of adipogenesis (Mozzetta et al., 2013; Marinkovic et al., 2019).

Giuliani et al. (2021) described dynamic SCA1 protein expression within FAPs, enabling a SCA1-driven micro-heterogeneity that influences their fate (Giuliani et al., 2021). SCA1^high^ FAPs exhibit greater potential for proliferation and adipogenesis than SCA^low^ FAPs. Additionally, adiponectin expression was highly upregulated in SCA1^high^ FAPs, suggesting that the autocrine factor adiponectin was responsible for their greater adipogenesis. Additionally, *Dpp4* expression was increased in SCA1^high^ FAPs (Giuliani et al., 2021). Several studies have demonstrated that immune cells play a crucial role in regulating FAP function and fate. Giuliani et al. (2021) showed that conditioned media from CD45^+^ leucocytes isolated from young dystrophic mice inhibited the adipogenic differentiation of SCA1^low^ and SCA1^high^ FAPs. However, leukocyte-conditioned media from old dystrophic mice was less effective at limiting SCA1^high^ FAP adipogenesis, which could help explain the appearance of IMAT in muscles of *mdx* mice, dystrophic patients and chronic diseased individuals (Fig. 2B).

The frequency of TCF7L2^medium/low^ differentiated FAPs increases concomitantly with ECM deposition and fibrosis in dystrophic and chronically inflamed muscle (Contreras et al., 2020). *In vitro* passaging of muscle FAPs causes a reduction in PDGFRα protein expression, likely resulting in myofibroblastic phenotypic changes that lead to the downregulation of FAPs as they lose their progenitor identity (Contreras et al., 2019c). This dynamic downregulation of PDGFRα and TCF7L2 can also be mediated by the pro-fibrotic TGF-β axis (Contreras et al. 2019a, 2020), which results in FAP activation under pathological fibrotic conditions and a loss of progenitor identity and myofibroblast transition. Interestingly, a recent study showed that fibrogenic potency of FAPs is partially regulated by ECM architecture and stiffness, and that FAPs can sense mechanic constraints via activation of the Yes-associated protein YAP (product of the *Yap1* gene) signaling (Loomis et al., 2022).

In human skeletal muscles from patients with T2DM, two FAP subpopulations with distinct phenotypic and molecular signatures were identified based on CD90 levels (Farup et al., 2021). A subset of Lin^−^/CD56^−^/CD82^−^/CD34^+^/CD90^+^ FAPs was associated with muscle fibrosis and insulin resistance in degenerative T2DM settings (Farup et al., 2021). Compared with CD90^−^ FAPs, CD90^+^ FAPs are larger, proliferate faster, express more ECM genes, and exhibit greater *in vitro* glycolytic flux and oxygen consumption (Farup et al., 2021). After PDGF-AA treatment, CD90^+^ FAPs exhibit enhanced fibrogenesis but reduced adipogenesis, whereas CD90^−^ FAPs exhibit enhanced adipogenesis (Farup et al., 2021). Additionally, FAPs isolated from muscle biopsies of patients with oculopharyngeal muscular dystrophy showed high proliferative and pro-fibrotic capacities in response to TGF-β pro-fibrotic signal and endothelin. Furthermore, in co-culture experiments, fibrotic FAPs impair the formation of myotubes by interfering with fusion of myogenic cells (Bensalah et al., 2022).

From a therapeutic perspective, metformin reduced proliferation, oxygen consumption, and adipogenesis but increased the glycolytic flux of CD90^+^ FAPs (Farup et al., 2021). Metformin is widely used as a frontline pharmacological approach for treating T2DM, mainly due to its glucose- and lipid-lowering effects, which reduce overall cardiovascular morbidity and mortality (Zhou et al., 2001; Gormsen et al., 2018). The effects of metformin are primarily attributed to the activation of AMP-dependent protein kinase (AMPK) (Rena et al., 2017). A recent study demonstrated that selective deletion of the AMPK subunit α1 in FAPs impaired muscle regeneration following injury and induced FAP fibrogenesis, suggesting metformin exerts an anti-fibrotic effect through AMPK signaling (Liu et al., 2023). Thus, identifying distinct FAP subsets based on their sensitivity to metformin could lead to the identification of a novel druggable cell-based target for reducing fibrosis and IMAT deposition in T2DM patients. Using mass cytometry, Petrilli et al. (2020) showed that CD90.2 identifies murine FAPs together with SCA1, CD34, PDGFRα, and vimentin (Petrilli et al., 2020) and that CD90 levels are associated with a pro-adipogenic Vcam1^+^ FAP subpopulation (Joe et al., 2010; Pisani et al., 2010b; Roberts et al., 2013).

In relation to obesity, Goodpaster et al. (2023) used scRNA-seq to profile 22 major subtypes of mesenchymal stromal cells from subcutaneous and visceral white adipose tissues, and skeletal muscle in response to HFD-induced obesity and exercise (Yang et al., 2022). Among these subtypes, seven muscle FAPs were identified: multipotent IPC_SkM, Cxcl14^+^ FAPs, proteoglycan-4^+^ FAPs (Prg4^+^), adipogenesis-regulating CD142^+^ FAPs, inflammatory post-injury-like FAPs, mesoangioblasts Alpl^+^ FAPs (MAB), and previously unreported MSC-derived pro-fibrotic Sca1^−^ FAPs. Among these cells, IPC_SkM and CD142^+^ FAPs were highly responsive to a HFD, exercise and the combination of these intervention, namely rescue intervention, (Yang et al., 2022). In single-cell transcriptomics, the rescue intervention led to 203 DEG versus 74 in exercise and 23 in HFD alone, mainly compromising IPC_SkM and FAP_CD142^+^ cells. In these cells, HFD upregulated the expression of genes involved in processes such as ECM organization, fat cell differentiation, inflammatory signaling pathways (IL-18 and cytokine, NF-κB), regulation of proteolysis, and circadian rhythm. On the other hand, training, with or without HFD, downregulated adipocyte differentiation preferentially in CD142^+^ FAPs, together with reducing ECM organization, inflammation-related pathways, while upregulating circadian pathways (Yang et al., 2022).

In the context of muscle atrophy and cachexia, the activation of the canonical WNT-β-catenin pathway is increased and leads to FAP-mediated fibrotic degeneration of muscle and myofiber atrophy (Kajabadi et al., 2023). Moreover, in transgenic mice engineered to harbor degradation-resistant β-catenin in FAPs, the constitutive accumulation of β-catenin led to fibrosis and rapid loss of muscle mass and function (Kajabadi et al., 2023). Indeed, activated FAPs had increased expression of atrogenic genes such as *Foxo1*, and secretion of the atrogenic factors of NOGGIN and ACTIVIN-A, a member of the TGF-β family ligands. Mechanistically, ACTIVIN-A binds to the type II activin receptor ACTVR2, potentially leading to increased SMAD2/3 activity in muscle fibers and ultimately, myofiber atrophy (Kajabadi et al., 2023).

Finally, a recent study by Fitzgerald et al. (2023) used single-nuclei RNAseq to identify three FAP subpopulations in the hip muscles from patients who underwent total hip replacement. These cells were MME^+^, GPC3^+^ and CD55^+^ FAPs (Fitzgerald et al., 2023), and the MME ^+^ subset differed from bona fide adipose tissue progenitors such as ICAM1^+^ and CD142^+^. MME^+^ FAPs expressed higher levels of *Cebpα* and *Pparγ* along with downregulated WNT ligand biogenesis, trafficking, and canonical WNT signaling (Fitzgerald et al., 2023). Notably, the MME ^+^ FAPs had a markedly greater adipogenic capacity under minimal *in vitro* adipogenesis (only insulin). Cell tracing experiments demonstrated that MME^+^ FAPs readily contribute to IMAT after GLY-induced muscle injury, and also express genes involved in vascular processes and angiogenesis. These characteristics resembled those of previously characterized LUM^+^ FAPs and muscle adipocytes (De Micheli et al., 2020b; Rubenstein et al., 2020). Interestingly, compared with MME^−^ FAPs, MME^+^ FAPs underwent selective apoptosis under regenerative conditions. In addition, at a later timepoint during regeneration, the basal number of MME^+^ FAPs was replenished by the transition of MME^−^ FAPs to MME^+^ FAPs (Fitzgerald et al., 2023). Finally, in muscles of the hip affected with OA that were chronically infiltrated with IMAT, there was a reduced number of MME^+^ FAPs, suggesting that FAP heterogeneity and composition are reduced, most likely due to their differentiation to fibro-fatty lineages (Fitzgerald et al., 2023).

## 4. Mechanisms regulating FAP activation, proliferation and survival

### 4.1. Immune cross-talk

The activation and proliferation of FAPs are primarily orchestrated by cell autonomous and cross-talk mechanisms with other stromal cells, endothelial cells, myofibers, and immune cells (Heredia et al., 2013; Mackey et al., 2017; Moratal et al., 2018; Mathes et al., 2021). Following muscle injury, infiltrating eosinophils secrete the type 2 innate cytokines interleukin-4 (IL-4) and interleukin-13 (IL-13), which induce the expression of IL-4 receptor-alpha (IL-4Rα) in FAPs. Activation of the IL-4Rα/STAT6 axis promotes FAP proliferation but inhibits adipogenic differentiation (Heredia et al., 2013). Moreover, IL-4-deficient mice exhibit increased IMAT in regenerating muscle, while treatment with IL-4 prevents the pro-adipogenic effects of adipogenic media on isolated FAPs (Heredia et al., 2013; Dong et al., 2014). Notably, initial immune infiltration and tissue granulation could be mediated by cytokines and chemokines secreted by FAPs one day after injury, indicating that FAPs are immunomodulatory cells (Scott et al., 2019; Theret et al., 2021).

### 4.2. FAP death is required for proper muscle regeneration

While the initial phase of muscle regeneration involves FAP expansion, the later phase requires freeing the space to replenish muscle with de novo myofibers. This requires eliminating a pool of the expanded FAPs after they accomplish their early pro-regenerative role, followed by the clearance of FAP debris. This progression relies partly on the cross-talk between FAPs and macrophages and the opposing effects of TNF-α and TGF-β signaling (Lemos et al., 2015; Tidball 2017). TNF-α signaling triggers FAP apoptosis, which relies on the upregulation of TNF-receptor superfamily members (*Tnfrsf*) and the downregulation of TGF-β signaling (Lemos et al., 2015; Fiore et al., 2016; Tidball 2017). Two studies have dissected this mechanism in fibroblasts (Wynes et al., 2011; Liu et al., 2017). First, TNF-α upregulated the expression of the Tnfrsf member cell surface death receptor Fas (Wynes et al., 2011). Second, upon TNF-α-induced activation, Fas trimerizes and translocates to lipid raft micro-domains at the plasma membrane, where it interacts with Thy1/CD90 and triggers apoptosis by blocking the anti-apoptotic effects of Bcl2 and Bcl-xL and by increasing caspase activation (Liu et al., 2017). Nonetheless, it is unknown whether CD90^−^ FAPs are resistant to apoptosis.

### 4.3. Pro-survival signals regulating apoptosis-dependent FAP clearance

The accumulation of apoptosis-resistant FAPs has been shown in a mouse model of chronic inflammatory myopathy (Saito et al., 2020). Moreover, anti-apoptotic FAPs reduce gene expression of *Tnfrsf*, including *Fas*; increase gene expression of all *TGF-β* isoforms; and upregulate *Tgfbr1* and *Tgfbr2* (Saito et al., 2020). In contrast, acute muscle injury triggered a senescence-like phenotype characterized by upregulation of *Cdkn2a* and *Trp53* expression and the histone variant *γH2A.X* (102). There is additional evidence for impaired FAP apoptosis in chronic conditions, as observed in *mdx* mice. FAPs isolated from dystrophic muscles exhibit a reduced apoptosis signaling pathway along with an upregulation of fibrogenic genes (Malecova et al., 2018). These findings support the notion that the microenvironment of chronic muscle disease favors anti-apoptotic FAP phenotypes that ultimately drive pathology.

Chronic diseases increase the proliferation of FAPs through chronic activation of mitogenic factors. TGF-β and PDGF ligands are potent mitogens that both induce FAP activation, proliferation, and differentiation (Lemos et al., 2015; Mueller et al., 2016; Contreras et al., 2019a; Mázala et al., 2020; Theret et al., 2021). These mitogens may be potentiated by interaction with the ECM glycoprotein thrombospondin-1 (TSP-1), product of the *Thsb1* gene (Belotti et al., 2016). TSP-1 expression is increased in the blood and muscle tissue of patients affected by brachio-cervical inflammatory myopathy and systemic sclerosis (BCIM-SSc), where muscle *Thbs1* expression is positively correlated with tissue fibrosis and infiltration of macrophages in the muscle (Suárez-Calvet et al., 2020). In addition, *in vitro* treatment with recombinant TSP-1, serum from BCIM-SSc patients or the supernatant of previously treated cells increased the proliferation of isolated human muscle-derived fibroblasts and TGF-β levels (Suárez-Calvet et al., 2020). Recent findings uncovered that FAPs secrete TSP-1 in response to the mechanical stress of surrounding ECM. This response is mediated by the transcriptional activation of YAP/TAZ (TAZ being the gene product of *Wwtr1*, encoding a transcriptional coactivator). In contrast to the known pro-fibrotic effect of TSP-1, this study describes a new role of FAP-secreted TSP-1, which acts by activating signaling by the CD47 receptor in MuSC, thereby driving their proliferation, and a pro-myogenic effect. This mechanism is critical to supply new myogenic nuclei for the enlargement of muscle fibers during load-related muscle hypertrophy (Kaneshige et al., 2022).

TGF-β is initially secreted in its latent, non-active pro-cytokine form, which is activated through a complex mechanism involving binding to TSP-1 and other factors (Crawford et al., 1998; Murphy-Ullrich and Suto 2018; Contreras et al., 2021b). TSP-1 binds to latent TGF-β through its type 1 repeats (KRFK sequence) and subsequently disrupts a conserved inhibitory sequence (LSKL) on TGF-β, thereby leading to TGF-β activation (Murphy-Ullrich and Suto 2018). Interestingly, hypertrophied visceral fat adipocytes have been identified as the primary source of circulating TSP-1 in individuals with insulin resistance or obesity and in HFD-fed mice (Varma et al., 2008; Buras et al., 2019). Elevated circulating levels of TSP-1 have also been associated with an increased risk of coronary and peripheral artery disease, T2DM, all-cause mortality, and adverse outcomes in chronic kidney failure patients (Smadja et al., 2011; Choi et al., 2012). Conversely, *Thbs1*-null mice are protected against obesity-induced metabolic alterations, largely by preventing fibrotic muscle degeneration (Inoue et al., 2013). In addition, dystrophic muscles from juvenile D2-*mdx* mice, a murine model of DMD, are characterized by a tonic increased in TGF-β and inflammatory responses to muscle damage, which is associated with worsening of histopathological changes (i.e., fibrosis and bone-like areas) and reduced regeneration (Mázala et al., 2023). Conversely, adult D2-*mdx* mice, despite exhibiting increased fibrosis, showed an improved muscle regeneration capacity. The differences between muscles of juvenile and adult dystrophic mice in terms of regenerative potential were associated with a predominant inflammatory niche in juvenile, characterized by increased expression of TNF-α, increased pro-inflammatory versus pro-regenerative macrophage activation, an impaired myogenic response, and elevated expression of pro-fibrotic markers such as Periostin and Osteopontin (Mázala et al., 2023). Interestingly, *in vitro* MuSC fusion and myotube formation were impaired when co-cultured with FAPs isolated from muscles of juvenile D2-*mdx* dystrophic mice (Mázala et al., 2023).

Secreted PDGFs are retained in the ECM, where they form complexes with ECM glycoproteins such as TSP-1. TSP-1 stabilizes PDGF-BB, thus facilitating its binding to membrane receptors on target cells (Tokunaga et al., 2008). PDGF signaling activation by PDGF-AA and PDGF-BB stimulates the proliferation and differentiation of FAPs (Uezumi et al., 2014; Mueller et al., 2016), which has been linked to pathological increases in PDGF ligand levels and PDGFRα overactivation, both of which are promoters of muscle fibrosis in chronically damaged muscles (Ieronimakis et al., 2016). Additionally, the cross-talk between PDGF and TGF-β modulates the fate of FAPs. Treatment of FAPs with TGF-β downregulates PDGFRα and induces fibrotic differentiation and migration while inhibiting FAP adipogenicity (Uezumi et al., 2014; Contreras et al., 2019a). Whether TSP-1 can mediate the cross-talk between TGF-β and PDGFRα warrants further investigation. Remarkably, inflammatory cells, endothelial cells, MuSCs, myofibers, and FAPs express *Pdgf* genes in adult muscles (Contreras et al., 2021a).

PDGF signaling regulates the activation, cell cycle, and fate of myogenic progenitors (Contreras et al., 2021a). Owing to this, the PDGF signaling pathway has emerged as a promising molecular target for promoting skeletal muscle regeneration and boosting tissue repair. From a clinical perspective, increased PDGF-AA serum concentrations correlate with several muscle functional tests in DMD patients (Alonso--Jiménez et al., 2021). Conversely, decreased levels of serum PDGF-AA at advanced stages of the disease are associated with muscle loss and weakness and increased IMAT (Alonso-Jiménez et al., 2021). These findings suggest that PDGF-AA can serve as a biomarker for monitoring disease progression in DMD patients.

PDGF-AA exerts a pro-migratory and proliferative effects on FAPs, a response that has been recently corroborated in human DMD FAPs (Fernández-Simón et al., 2022). Proteomic analysis of these cells revealed that upregulation of the RhoA/ROCK2 axis modulates PDGF-AA-promoted migration, proliferation, and fibrogenic differentiation (Fernández-Simón et al., 2022). This mechanism may be relevant for preventing FAP accumulation, fibrogenic differentiation, and subsequent fibrotic muscle degeneration observed in T2DM patients (Farup et al., 2021). To test this hypothesis, Fernández-Simón et al. (2022) treated DBA/2 J-*mdx* mice with fasudil, a pan-Rho-kinase inhibitor, and found reduced FAP numbers, decreased Collagen I staining, and increased grip strength in the treated animals (Fernández-Simón et al., 2022). These results demonstrated that the PDGF-PDGFR-RhoA/ROCK2 axis is a targetable pathway through which FAPs enhance muscle regeneration in disease settings. However, further studies are needed to fully understand the therapeutic potential of manipulating this pathway.

## 5. Cellular and molecular regulatory mechanisms controlling FAP adipogenesis

Under homeostatic conditions, several processes work together to restrain FAP adipogenesis while maintaining its steady state. These processes include, but are not restricted to, cell-autonomous signals such as WNT/β-catenin, NOTCH, Annexin-A2 (AnxA2), and Hedgehog (Hh) (Defour et al., 2017; Kopinke et al., 2017; Marinkovic et al., 2019). Understanding how FAP adipogenic commitment and adipocyte maturation regulate chronic diseases could lead to new treatments for muscle degeneration. Therefore, in this review, we have explored the role of these cues in both normal and diseased conditions.

### 5.1. WNT/β-catenin signaling and TCF/LEF transcription factors

FAPs are relevant sources of WNT ligands in muscle (Reggio et al., 2020b). Proliferative FAPs have a high content of cytoplasmic β-catenin, but adipogenic induction reduces the β-catenin protein level, while the β-catenin mRNA level remains stable. This suggests that post-transcriptional degradation of β-catenin is involved in adipocyte commitment (Reggio et al., 2020b). In addition to FAPs, macrophages produce a significant amount of WNT ligands in skeletal muscle in response to acute injury (Tusavitz et al., 2020). Additionally, muscle fibers actively secrete WNT4A, which helps maintain MuSC quiescence (Eliazer et al., 2019).

WNT signaling activation is well known to inhibit adipogenesis (Ross et al., 2000; Bennett et al., 2002; Longo et al., 2004). Overexpression of WNT1, a phosphorylation-resistant β-catenin, or inhibition of β-catenin phosphorylation with lithium chloride prevented the adipogenic differentiation of 3T3-L1 pre-adipocytes (Ross et al., 2000).

In murine models, selective overexpression of WNT10B from the fatty-acid binding protein 4 (FABP4) promoter repressed the expression of the adipogenic program in adipocytes, thus impairing adipose tissue development and preventing obesity in mice (Longo et al., 2004; Wright et al., 2007). In a study by Fu et al. (2023), the WNT7A protein inhibited FAP adipogenesis *in vitro* and reduced adipocyte formation in response to GLY-induced injury without affecting myofiber size. Furthermore, a positive feedback loop was suggested in which WNT-Rho-YAP/TAZ upregulates canonical WNT signaling in activated FAPs (Fu et al., 2023), supporting the idea of manipulating WNT signaling as a way to reduce IMAT.

WNT TCF/LEF transcription factors also play a key role in adipocyte cell fate determination. Early evidence showed that overexpression of a dominant-negative form of TCF7L2 caused pre-adipocytes differentiation to adipocytes (Ross et al., 2000). In terms of adipogenic commitment, TCF7L1, another TCF/LEF member, acts as a transcriptional repressor that elicits adipogenic differentiation (Cristancho et al., 2011). Interestingly, TCF7L1 inhibited adipogenic differentiation, whereas its overexpression led to the opposite effects (Cristancho et al., 2011). Although *Tcf7l1* is highly expressed in FAPs (Contreras et al., 2020), the role of TCF7L1 in FAPs has not been addressed. Regarding TCF7L2, Geoghegan et al. (2019) showed that adipose-specific *Tcf7l2* knockout mice exhibited altered glucose metabolism, insulin responsiveness, weight gain, and increased adipose tissue mass in response to a HFD (Geoghegan et al., 2019). Thus, TCF7L2 regulates energy consumption, insulin homeostasis, lipolysis and lipogenesis in adipose tissue (Geoghegan et al., 2019). Notably, *Tcf7l2* deletion in pre-adipocytes enhanced adipocyte differentiation and lipid accumulation (Geoghegan et al., 2019). Using RNA-seq and TCF7L2 ChIP-seq integration analysis of adipocytes, TCF7L2 was shown to occupy and regulate genes involved in metabolic homeostasis and the cell cycle. These findings showed that the WNT effector TCF7L2 modulates adipocyte hypertrophy and links this phenotype to genome-wide occupancy of metabolic-associated genes. However, the specific mechanisms by which TCF7L2 regulates lipid and metabolic genes have yet to be determined, although previous evidence has shown that co-factors regulate the ability of TCF7L2 to bind DNA and regulate gene expression (Norton et al., 2014).

In limb muscles, the loss of *Tcf7l2* in FAPs reduced the expression of myosin heavy chain type I and embryonic myosin, thus affecting the formation of large multinucleated myofibers (Mathew et al., 2011). We demonstrated that TGF-β-mediated TCF7L2 downregulation coincides with fibrogenic differentiation of FAPs (Contreras et al., 2020). TGF-β promoted TCF7L2 protein degradation via the ubiquitin‒proteasome system and repressed *Tcf7l2* gene expression through histone deacetylases (Contreras et al., 2020). In parallel, selective inhibition of GSK-3, an upstream β-catenin kinase, reversed adipogenic differentiation of FAPs and IMAT degeneration in *mdx* mice (Reggio et al., 2020b), demonstrating that β-catenin mediates FAP adipogenesis.

Si et al. (2006) showed that the matricellular protein cysteine-rich protein 61 (Cyr61, also known as CCN1) was a WNT/β-catenin target and that moreover, CCN1 was required for WNT-mediated regulation of mesenchymal stromal cell fate (Si et al., 2006). *Ccn1* silencing in bone marrow stromal cells and C3H10T1/2 mesenchymal cells reduced β-catenin and TCF7L2 protein levels in the nucleus and activated mTORC1, thereby driving adipogenic differentiation. Conversely, CCN1 overexpression inhibited adipogenic differentiation (Yang et al., 2018). However, elevated CCN1 levels in muscle and serum have been associated with increased FAP adipogenic differentiation in humans and in mice with chronic kidney disease (Hu et al., 2019). Additionally, increased serum levels of Dickkopf-related protein 1 (DKK1), a known inhibitor of WNT signaling, have been associated with increased IMAT deposition in individuals with obesity and insulin resistance (Ali et al., 2019).

Reciprocal regulation occurs between adipogenic signals and WNT-mediated adipogenesis repression. Mechanistically, pharmacological induction or conditional expression of CCAAT/enhancer-binding protein alpha (C/EBPα) suppresses nuclear WNT/β-catenin-TCF/LEF signaling during adipogenesis of 3T3-L1 pre-adipocytes (Moldes et al., 2003). This decrease in the nuclear level of β-catenin occurred in a dose-dependent manner following ligand-induced PPARγ activation (Moldes et al., 2003). The inhibitory effect of PPARγ on WNT/β-catenin-TCF/LEF signaling has been attributed to TLE3, a potent adipogenic factor that facilitates PPARγ activity on its target genes while repressing TCF/LEF activity (Villanueva et al., 2011).

In contrast to the anti-adipogenic effect of canonical WNT/β-catenin signaling, activation of the non-canonical pathway seems to induce adipogenesis. Keats et al. (2014) reported that the adipogenic effect of a high-glucose environment was mediated by autogenous activation of the non-canonical WNT11/Ca^2+^/protein kinase C signaling. High glucose triggered adipogenesis by upregulating C/EBPβ and C/EBPδ. This effect was mediated by increased *Wnt11* expression and subsequent autocrine WNT11-mediated activation of non-canonical PKC signaling (Keats et al., 2014). Therefore, the balance between WNT canonical and non-canonical mechanisms that regulate FAP adipogenesis has yet to be fully elucidated.

### 5.2. Notch signaling

NOTCH signaling regulates FAP adipogenesis in an autocrine/juxtacrine manner (Marinkovic et al., 2019). Treatment with the γ-secretase inhibitor DAPT, which inhibits proteolytic cleavage of NICD, abrogates the anti-adipogenic effect of NOTCH signaling in FAPs cultured in DLL1-coated plates. Similarly, knockdown of the NOTCH2 receptor in FAPs increased adipogenesis and PPARγ expression (Marinkovic et al., 2019).

MuSCs and myotubes inhibit FAP adipogenic differentiation *in vitro* when these cells are co-cultured. This effect is mediated by NOTCH signaling activation, as exposure to DAPT increased FAP adipogenesis, lipid droplet accumulation and PPARγ expression (Marinkovic et al., 2019). In addition, mice treated with intraperitoneal injections of DAPT following CTX-induced muscle injury developed IMAT (Marinkovic et al., 2019). Taken together, these findings revealed the critical role of NOTCH signaling in limiting the adipogenic potency of FAPs. Intriguingly, FAPs isolated from dystrophic mice were insensitive to NOTCH signaling (Marinkovic et al., 2019). This desensitization was attributed to proteomic changes, including downregulation of components of the SWI/SNF chromatin-remodeling complex, upregulation of CDK5, an upstream kinase that increases PPARγ transcriptional activity, and upregulation of the retinoic X receptor alpha, which forms a heterodimer with PPARγ to drive adipogenesis (Marinkovic et al., 2019). Remarkably, signals associated with the inflammatory response to muscle injury, mainly TNF-α and NFκB, potentiate NOTCH signaling and increase its anti-adipogenic effect (Marinkovic et al., 2019). These findings corroborate prior studies suggesting that FAPs from uninjured and dystrophic muscle exist in different cell states.

### 5.3. Annexin-A2

Limb girdle muscular dystrophy type 2 B (LGMB2B) is caused by disease-causing variants in the *DYSFERLIN* gene and characterized by late-onset muscle weakness and muscle wasting associated with IMAT (Grounds et al., 2014; Hogarth et al., 2019). Dysferlin is a membrane-associated protein involved in Ca^2+^-dependent sarcolemmal stability, vesicle trafficking, and membrane fusion (Grounds et al., 2014). Thus, the absence of dysferlin impairs sarcolemmal repair and disrupts Ca^2+^ homeostasis, leading to chronic inflammation, muscle degeneration, and gradual IMAT development (Grounds et al., 2014; Defour et al., 2017; Hogarth et al., 2019). The appearance of IMAT in the dysferlinopathic muscles of mouse models has been associated with the accumulation of Annexin-A2 (AnxA2), a protein that interacts with Dysferlin to facilitate sarcolemma repair after injury (Defour et al., 2017; Agarwal et al., 2019; Hogarth et al., 2019). As a Ca^2+^-dependent phospholipid-binding protein with intracellular and extracellular roles, intracellular AnxA2 interacts with dysferlin and actin to facilitate plasma membrane repair (McDade et al., 2014). Extracellular AnxA2 is produced by several cell types, including myofibers, monocytes and macrophages, and is secreted as both a soluble protein and a membrane-bound protein via two mechanisms: oxidative stress-mediated p38 MAPK activation and insulin/IGF-1 receptor activation (Zhao et al., 2003; Brownstein et al., 2004; Kita et al., 2016).

Interstitial AnxA2 levels positively correlate with clinical severity and myodegeneration in Dysferlin-deficient muscles (Cagliani et al., 2005; Hogarth et al., 2019). In dysferlinopathic mice, the severity of symptoms, inflammation and adipogenesis are reduced upon *AnxA2* deletion, despite poor sarcolemmal repair (Defour et al., 2017). Remarkably, Hogarth and colleagues identified FAPs as the driving cells of fatty degeneration in the B6A/J mouse, a murine model of LGMB2B (Hogarth et al., 2019). FAPs accumulate and differentiate into adipocytes in dysferlin-deficient old mice after repeated rounds of injury-regeneration. FAPs and macrophages are enriched in interstitial regions where AnxA2 levels increase, suggesting that both cell types are recruited to the injured area in response to increased AnxA2 (Hogarth et al., 2019). In fact, compared with dysferlin-deficient mice, double knockout Dysferlin/AnxA2 mice (A2-B6A/J) exhibited reversal of macrophage infiltration and FAP-driven fatty degeneration (Hogarth et al., 2019). Remarkably, *in vitro* spontaneous adipogenesis is increased in dysferlinopathic FAPs but reduced in A2-B6A/J FAPs. Furthermore, treatment of dysferlinopathic FAPs with 100 μM AnxA2 drastically increased spontaneous adipogenic differentiation, and notexin-induced injury in the presence of exogenous AnxA2 increased the number of FAPs and adipocytes. Hence, in individuals with Dysferlin deficiency, impaired membrane repair and chronic inflammation create a loop in which damaged muscle fibers amplify AnxA2 production, which further fuels adipocyte accumulation, leading to excessive IMAT deposits in individuals with dysferlinopathy.

### 5.4. Ciliary hedgehog signaling

Another pathway that controls the adipogenic fate of FAPs is the Hedgehog (Hh) pathway (Kopinke et al., 2017). Hh signaling relies on microtubule-based ‘antennae’ called primary cilia to receive and interpret external cues (Goetz and Anderson 2010; Anvarian et al., 2019; Kopinke et al., 2021). Ciliary-controlled signaling events are crucial for tissue patterning because they mediate individual cell fate decisions and coordinate cell‒cell communication. In addition to Hh signaling, multiple signaling pathways, such as the TGF-β, PDGF, WNT, GPCR and NOTCH signaling pathways, have been shown to utilize cilia for their functions (Goetz and Anderson 2010; Arrighi et al., 2017; Kopinke et al. 2017, 2021; Malicki and Johnson 2017; Anvarian et al., 2019; Hilgendorf et al., 2019). Hh signaling initiates with the production and secretion of Hh ligands: Sonic (Shh), Indian (Ihh) and Desert (Dhh) hedgehog proteins. In the off-state, primary cilia repress Hh target genes such as *Gli1* and *Ptch1* via the formation of the transcriptional repressor GLI3 (Liu et al., 2005). The binding of a Hh ligand to the receptor Patched-1 (PTCH1) allows the G protein–coupled receptor–like transmembrane protein Smoothened (Smo) to enter the cilium (Liu et al., 2005; Ruiz i Altaba 2011; Kopinke et al., 2017). Once inside the cilium, Smo activity promotes the formation of the transcriptional activator GLI2 and prevents GLI3 repressor formation (Liu et al., 2005).

Within skeletal muscle, FAPs are the major ciliated cell type (Kopinke et al., 2017). During muscle repair, FAP ciliation dynamically changes. While few FAPs are ciliated prior to injury, their ciliation frequently increases immediately after acute injury before returning to pre-injury levels (Kopinke et al., 2017). Interestingly, FAP cilia are remodeled and lost early during differentiation into adipocytes (Kopinke et al., 2017; Hilgendorf et al., 2019). To probe the function of FAP cilia, we conditionally deleted intraflagellar transport 88 (Ift88), a gene required for ciliogenesis and ciliary maintenance, specifically from FAPs. Loss of FAP cilia prevents the differentiation of FAPs into Plin1^+^/FABP4^+^ adipocytes following acute muscle injury (Kopinke et al., 2017). Similarly, the removal of cilia in FAPs drastically reduced FAP adipogenesis and, consequently, IMAT infiltration in dystrophic muscles of *mdx* mice (Fig. 2B). In parallel, the loss of FAP cilia enhanced muscle regeneration and increased myofiber size, thus preserving muscle regenerative capacity in mice after GLY-induced muscle injury as well as in muscles of dystrophic *mdx* mice.

Mechanistically, we found that FAP cilia loss increased the expression of Hh target genes due to loss of the GLI3 repressor, resulting in Hh derepression. This low-level Hh activation upon loss of cilia was sufficient to repress IMAT formation. Similarly, we found that directly activating Hh signaling via conditional deletion of *Ptch1* in FAPs also repressed the ability of FAPs to differentiate into adipocytes (Kopinke et al., 2017). Likewise, the activation of Smo with a small-molecule agonist (SAG) replicated the upstream anti-adipogenic effects of Hh signaling activation and cilia removal. We further determined that ciliary Hh signaling promotes adipogenesis non-cell-autonomously via upregulation of the expression of the secreted anti-adipogenic protein tissue inhibitor of metalloproteinase 3 (TIMP3). Loss of FAP cilia and activation of Hh signaling via removal of *Ptch1* upregulated *Timp3* expression (Kopinke et al., 2017). TIMP-3 is involved in the regulation of essential early inducers of adipogenesis, including *C/ebpβ,* and its down-regulation is mandatory for proper implementation of the adipocyte differentiation program (Bernot et al., 2010). Furthermore, TIMP3 modulates the shedding of the NOTCH ligand DLK1 and ECM remodeling via collagen 1 degradation and inhibition of the metalloproteinases ADAM17 and MMP14 to allow adipose expansion (i.e., adipocyte hypertrophy) (Fenech et al., 2015). The anti-adipogenic effect of ciliary Hh signaling activation through TIMP3 relies on its effect on MMP14 activity (Kopinke et al., 2017). Pharmacological inhibition of metalloproteinases with batimastat or specific inhibition of MMP14 by NSC405020 mimicked the effects of TIMP3 and robustly inhibited adipogenesis of FAPs *in vitro* and *in vivo* after GLY-induced injury (Kopinke et al., 2017).

Our recent findings demonstrated that DHH, which is sensed by FAPs, is the key Hh ligand involved in muscle regeneration. Genetic loss of *Dhh* resulted in accelerated IMAT formation due to repression of TIMP3 (Norris et al., 2023). Moreover, myofiber regeneration was severely impacted by the absence of Hh activity due to the lack of induction of GDF10 (Bmp3b), a potent myogenic factor (Uezumi et al., 2021), within FAPs. Notably, the adipo-regulatory CD142^+^ FAPs subpopulation inhibits adipogenesis via the secretion of GDF10. Interestingly, CD142^+^ FAPs are almost completely lost in dystrophic mice, thus leading to increased adipogenesis (Camps et al., 2020).

Together, our findings reveal an undescribed role for cilia-mediated Hh signaling in FAP adipogenesis and IMAT accumulation and identify Hh signaling as an endogenous adipogenic brake. In this context, a previous study provided further evidence that FAPs are the major Hh responders. Yao et al. (2021) described a subpopulation of Gli1^+^ FAPs (Gli1 is a direct Hh target) that preferentially expands upon injury-mediated Hh signaling activation (Yao et al., 2021). These cells retain greater clonogenic capabilities at the expense of reduced adipogenic differentiation capacity than Gli1^−^ FAPs. Remarkably, both the depletion of Gli1^+^ FAPs and the pharmacological inhibition of Hh signaling by GANT61 led to the increased formation of Perilipin^+^ adipocytes after GLY-induced injury (Yao et al., 2021). In summary, pharmacological strategies aimed at reducing fatty degeneration and improving the regeneration of skeletal muscle should consider the modulation of ciliary Hh and its effect on TIMP3-mediated MMP14 activity.

### 5.5. FAP dynamics and adipogenic potency in neuromuscular trauma and disease

Sciatic nerve denervation causes pronounced muscle atrophy, ECM deposition, and accumulation of FAPs (Contreras et al., 2016; Madaro et al., 2018; Rebolledo et al., 2019). Similarly, FAPs expand in response to chronic neurodegenerative diseases such as amyotrophic lateral sclerosis (ALS). Specifically, the loss of neuromuscular junction integrity caused by denervation or ALS has been related to the activation, expansion, and differentiation of FAPs and the accumulation of fibro-fatty tissues (Contreras et al. 2016, 2019c; Gonzalez et al., 2017; Madaro et al., 2018). Thus, excessive activation and accumulation of FAPs may contribute to the progression of muscle wasting (Theret et al., 2021). FAPs from denervated muscles exhibit pro-fibrotic properties and express genes associated with inflammation and pro-atrophic and catabolic factors (Madaro et al., 2018). For instance, persistent secretion of IL-6 and subsequent IL-6-mediated activation of STAT3 signaling in FAPs from denervated muscles induced muscle fiber atrophy and fibrosis (Madaro et al., 2018). *In vivo* blockade of IL-6 signaling via a neutralizing antibody or a selective STAT3 inhibitor prevents FAP-mediated muscle atrophy and fibrosis (Madaro et al., 2018). Remarkably, the PDGFRα^+^/CD34^+^/Sca-1^−^ FAP subtype is particularly prone to upregulating IL-6 secretion and initiating an oxidative stress response that contributes to denervation-induced muscle atrophy (Yang et al., 2021). These findings highlight the importance of IL-6 signaling in FAP-mediated muscle loss and suggest that targeting this pathway has therapeutic potential for muscle wasting diseases.

Two recent studies utilized single-cell RNA sequencing (scRNA-seq) to investigate the response of muscle-resident cells to transection of the sciatic nerve and identified two cell populations responsive to denervation: muscle glial cells (likely activated Schwann cells) and FAP-derived activated fibroblasts (*Pdgfra*^low^ and *Sca1*^low^) (Nicoletti et al., 2020; Proietti et al., 2021). Both cell populations quickly respond to denervation by expanding their numbers and upregulating transcription (Nicoletti et al., 2023). Interestingly, Schwann cells and FAPs closely interact in muscle nerve- and neuromuscular junction (NMJ)-associated niches, where FAPs regulate Schwann cell dynamics and homeostasis (Theret et al., 2021; Uezumi et al., 2021). These and previous studies reporting FAP and fibroblast activation after denervation (Contreras et al., 2016; Gonzalez et al., 2017; Madaro et al., 2018; Rebolledo et al., 2019), indicate that denervation drives crosstalk between activated glial cells and FAPs (Wei et al., 2021). Overall, these studies revealed the cellular responses to denervation in muscle, highlighting the complex interplay between nerve cells and FAPs, which promotes a non-regenerative environment in response to denervation-induced NMJ catabolic stimuli.

Acute myotrauma leads to the early expansion of non-resident and muscle-resident immune cells, whereas denervation does not immediately induce significant immune cell infiltration (Madaro et al., 2018; Nicoletti et al., 2020; Oprescu et al., 2020; Theret et al., 2021; Wei et al., 2021). These findings were supported by a study in which single-nucleus RNA sequencing was utilized to decode the transcriptome of the gastrocnemius muscle after sciatic nerve denervation (Lin et al., 2021). In this context, C1, C2, and C3 FAP clusters were described with transcriptional trajectories that expanded to form three main branches, with C2 forming the fibrotic branch and C3 the adipogenic branch (Lin et al., 2021), corroborating that the FAP transcriptome responds to denervation, as previously shown (Madaro et al., 2018). Using gene set enrichment analysis, the authors found that denervation downregulated myogenesis, hypoxia, and metabolism-related gene expression in all the FAP cells, with differentially upregulated gene sets in each of the three FAP clusters. For example, denervated C1 FAPs were associated with apoptotic p53 gene sets, C2 FAPs were associated with fibrosis and angiogenesis, and C3 FAPs were associated with adipogenesis. The denervation-mediated transcriptional changes in FAPs could be driven by specific regulons, such as TCF7L2 and SREBF1, which are activated in fibrotic C2 and adipogenic C3 FAPs. Our previous studies demonstrated that TCF7L2-expressing cells expand in response to sciatic denervation and in ALS-affected muscles (Contreras et al., 2016; Gonzalez et al., 2017). We also showed that the WNT-associated TCF7L2 transcription factor is downregulated by pro-fibrotic TGF-β signaling in FAPs and stromal cells. This provides a new cross-talk mechanism between TGF-β and WNT signaling pathways that may regulate fibrogenic fate and tissue fibrosis (Contreras et al., 2019a).

Interestingly, the C3 cluster was similar to that of Dpp4^+^ FAPs (Scott et al., 2019; Oprescu et al., 2020). Dpp4^+^ stromal cells have been shown to contribute to basal and de novo diet-induced adipogenesis in adipose tissue and bone marrow (Ambrosi et al., 2017; Merrick et al., 2019), suggesting that Dpp4-expressing FAPs could be adipogenic progenitors in skeletal muscles. However, recent evidence challenges this role, particularly in response to a HFD (Takada et al., 2022). Collectively, these findings suggest that tissue-resident macrophages, glial cells, and FAPs actively engage in cross-talk to modulate denervation onset, development, and degenerative outcomes.

### 5.6. Muscle disuse and deconditioning as triggers for FAP activation and IMAT accumulation

Reduced physical activity is associated with a loss of muscle mass, which is a strong determinant of IMAT accumulation, predicting up to 50% of its variance (Manini et al., 2007; Akima et al., 2015). Decreased muscle activity or deconditioning could cause muscle atrophy and FAP accumulation, even when neuromuscular function is preserved (Manini et al., 2007; Pagano et al. 2015, 2018). Dry immersion (to reproduce the effects of microgravity) promoted muscle wasting (~11% myofiber area reduction) in lean adult patients, accompanied by IMAT expansion and ~50% elevated protein levels of FAP and FAP-derived adipocyte markers (Pagano et al., 2018). Additionally, immobilization upregulated two senescence-associated genes in murine FAPs, *Il-1β* and *Cdkn2a*, suggesting that FAP senescence may play a novel role in the development of disuse muscle atrophy (Parker et al., 2022).

**Fig. 3.**
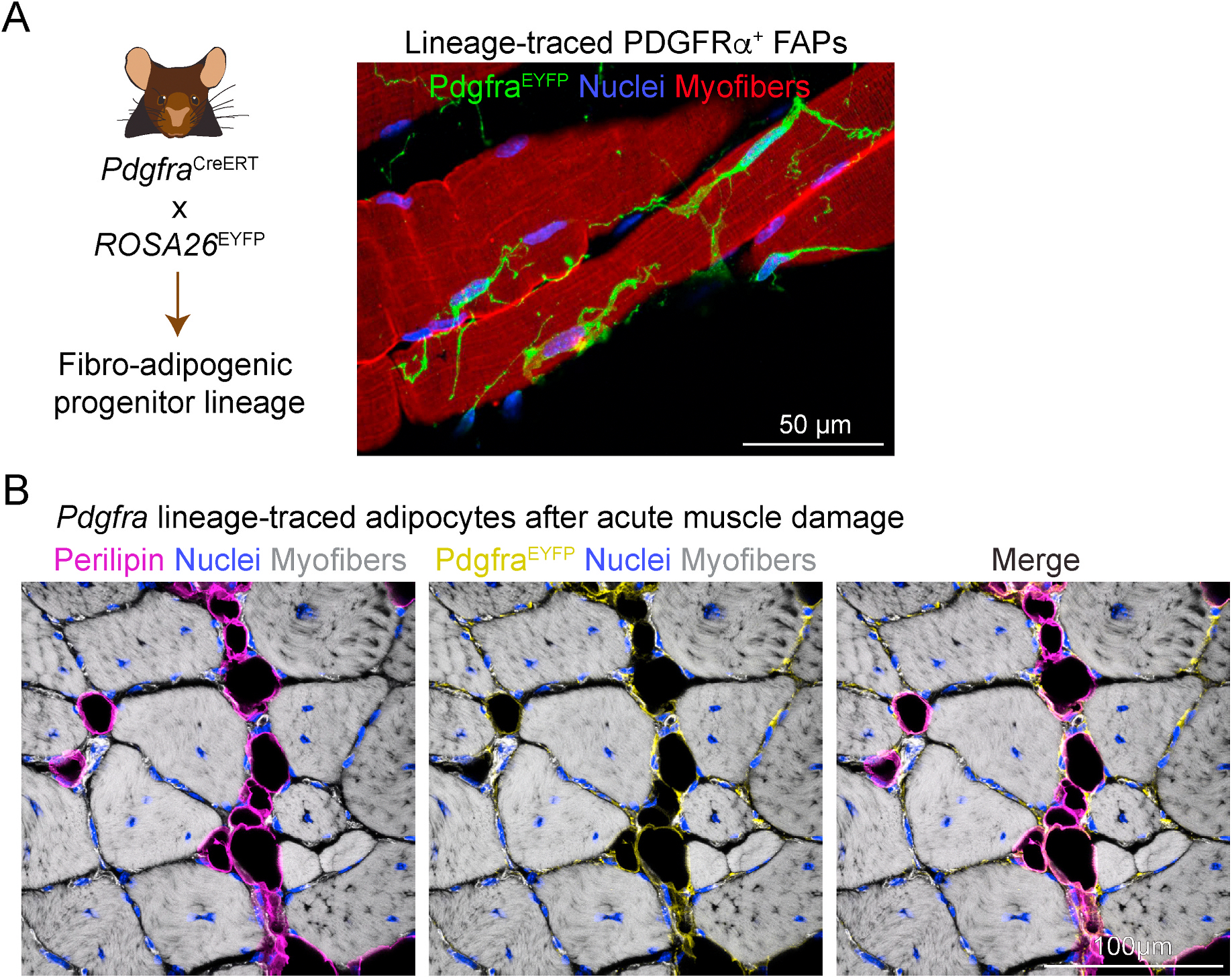
Adipocyte cell formation and IMAT deposition *in vivo* from muscle-resident PDGFRα^+^ fibro-adipogenic progenitors. (A) *Pdgfrα* lineage tracing shows the complex cell structures and protrusions of muscle-resident FAPs and their close steady-state contact with healthy myofibers. (B) Lineage tracing reveals that IMAT-associated adipocytes (perilipin^+^) are derived from PDGFRα-expressing FAPs.

Diet-induced obesity has been associated with myofiber atrophy and sarcopenia, currently known as sarcopenic obesity (Song et al., 2004; Biolo et al., 2014; Fellner et al., 2014; Collins et al. 2016a, 2016b; Mogi et al., 2016; Buras et al., 2019). Genetic lineage tracing experiments using Pdgfrα-CreERT2 mice crossed with R26R-EYFP mice confirmed that HFD-induced IMAT was composed of adipocytes derived from FAPs (Takada et al., 2022), similar to what we and others have observed in multiple settings (Kopinke et al., 2017; Stumm et al., 2018; Hogarth et al., 2019) (Fig. 3). In the context of diet-induced obesity, muscles are characterized by a reduced myofiber cross-sectional area and a transient accumulation of senescent FAPs (Takada et al., 2022). Senescent FAPs in HFD-fed mice exhibit upregulation of genes related to the PPARγ signaling pathway and adipogenesis, such as *perilipin-1* (PLIN1), *Fabp4*, and *Cd36* (Takada et al., 2022). These results suggest that a HFD could influence the obesogenic microenvironment and therefore promote muscle atrophy by inducing some FAPs to become adipogenic.

In relation to inflammation, inflammation-mediated IL-1β secretion by macrophages may inhibit FAP-derived adipogenesis, whereas macrophage depletion via clodronate-loaded liposome treatment increases IMAT deposition after CTX-induced injury (Marinkovic et al., 2019). Another study showed that macrophages activated with IL-1β-conditioned media secreted unidentified factors that inhibited FAP adipogenesis via paracrine signaling. Moreover, both the IL-1α and IL-1β isoforms effectively suppress FAP adipogenesis while not affecting their proliferation (Vumbaca et al., 2021). Conversely, macrophage activation via exposure to the anti-inflammatory cytokine IL-4 promoted FAP adipogenesis (Moratal et al., 2018). These results reflect the complex regulation of FAP activity in response to injury, inflammation and disease. FAPs may have evolved to self-regulate their cellular states by secreting both anti- and pro-adipogenic factors in a balanced manner, potentially as a safeguard mechanism. There are data that support this concept. Injury-induced intronic polyadenylation of *Pdgfra* results in the transcription of different *Pdgfra* isoforms, including one with a truncated kinase domain. These isoforms regulate FAP activity and may determine the fate of FAP proteins, thus impacting the development of muscle fibrosis (Mueller et al., 2016). Further research is warranted to unravel the complex and dynamic interplay between FAPs, macrophages, and cytokines in the context of muscle homeostasis and inactivity.

When muscle activity is chronically reduced, for example, due to exercise detraining or prolonged immobilization, the factors promoting IMAT formation become dominant, a situation that is exacerbated by aging (Taaffe et al., 2009; Popadic Gacesa et al., 2011; Sertie et al., 2019; Mathes et al., 2021). In this scenario, the initial recruitment of pro-regenerative FAPs and their secretion of pro-myogenic factors may no longer counteract the negative effects of muscle inactivity, thus leading to abnormal fibrogenic and adipogenic programs. In support of this, healthy young adults who underwent four weeks of unilateral limb suspension experienced a concurrent 8% decrease in muscle mass and a 17% increase in IMAT content in the suspended limb, as determined by MRI (Manini et al., 2007). Notably, muscle loss accounted for 26% of the variance in IMAT accumulation (Manini et al., 2007).

In contrast to the causative role of muscle deconditioning in IMAT accumulation, studies in mice in which hindlimb unloading was used after acute GLY-induced muscle injury showed that by reducing muscle use, FAP accumulation and IMAT formation were prevented in regenerating muscles (Pagano et al. 2015, 2019). These effects were attributed to the maintenance of an inflammatory state post-injury that prevented FAP expansion by maintaining active TNF-α-mediated apoptotic signaling and reducing pro-proliferative TGF-β levels (Pagano et al., 2019). Accordingly, treating GLY-injured mice with decorin, an ECM and collagen-associated small leucine-rich proteoglycan that inhibits TGF-β, prevents IMAT accumulation (Pagano et al., 2019).

**Fig. 4.**
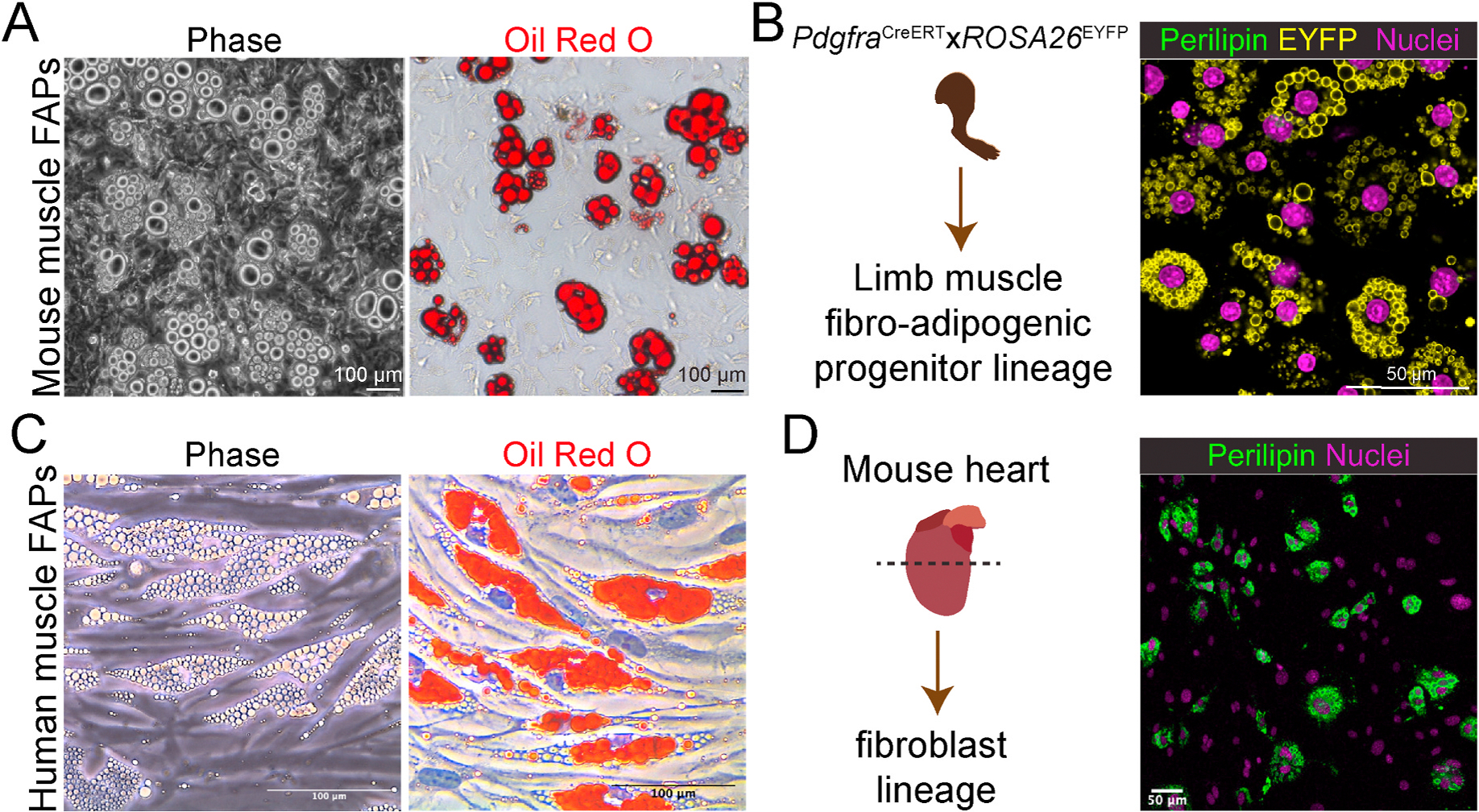
Formation of lipid droplet-rich adipocytes from fibro-adipogenic progenitors in cell culture. (A) Mouse muscle FAPs can be readily differentiated into functional adipocytes containing lipid droplets, as stained with Oil Red O. (B) PDGFRα lineage tracing shows that FAPs can generate adipocytes in culture with lipid droplets of different sizes and abundance. (C) Human muscle FAPs can also be differentiated into adipocytes that heavily accumulate lipid droplets. (D) Ventricle-resident fibroblasts can also generate perilipin^+^ adipocytes *in vitro*, although their adipogenic potential is known to be restrained *in vivo*.

These findings suggest the occurrence of intricate, intercellular interactions between FAPs, immune cells, MuSCs, and mature myofibers that govern muscle mass maintenance and IMAT development. However, how myofibers communicate their activity state to nearby FAPs in the interstitial space is unknown. Despite being located in different compartments separated by the basal lamina, myofibers and MuSCs can inhibit FAP adipogenesis via cell-to-cell contact through NOTCH, retinoic acid signaling and Anx2 after muscle injury-induced basal lamina disruptions (Hogarth et al., 2019; Marinkovic et al., 2019). Reinforcing the importance of direct cell contact, neither conditioned media from differentiating myoblasts nor a transwell co-culture system (allowing media exchange but not direct contact) could block FAP adipogenesis, as previously described (Uezumi et al., 2010). However, whether uncharacterized secreted factors released by myofibers (i.e., myokines) are also involved in FAP adipogenesis is unknown. Whether physical activity at the organismal level prevents the formation of IMAT by altering the phenotypical characteristics of FAPs via the release of exercise-induced myokines remains to be explored. Notably, combined advances in isolated FAP cell culture and optimization of differentiation media have enabled the generation of functional adipocytes from animal models and human FAPs (Johnson et al., 2022), establishing this approach as the gold standard for evaluating adipogenesis and FAP cell fate (Fig. 4A–C).

### 5.7. FAP adipogenesis and IMAT accumulation in aging and unhealthy aging

One of the most important physiological challenges affecting FAP function is aging. The proliferation and adipogenic capacities of muscle FAPs are significantly affected by aging. An early study by Mozzetta et al. (2013) revealed the enhanced adipogenic capacity of FAPs from the muscles of young *mdx* mice, which was reduced in FAPs from old *mdx* mice. Young FAPs also exert potent pro-myogenic effects via follistatin secretion on the activation of MuSCs and subsequent formation of myotubes (Mozzetta et al., 2013). Conversely, these effects were markedly reduced in aged *mdx*-FAPs. Interestingly, treatment with the histone deacetylase pan-inhibitor trichostatin A (TSA) reduced adipogenic potency while increasing the pro-myogenic effects of young *mdx*-FAPs; however, these effects were lost in aged *mdx*-FAPs. When young, pro-regenerative *mdx*-FAPs were transplanted into the muscles of old *mdx* mice, the capacity of TSA to promote regeneration was rescued (Mozzetta et al., 2013). This rescuing effect of the pan-HDAC inhibitor on the pro-regenerative capacity of aged FAPs suggested that over time, with the increase in degeneration/regeneration cycles, FAP properties change, coinciding with the accumulation of epigenetic marks on chromatin that can permanently impact the transcriptional output of FAPs, affecting their functional properties. In 15-month-old dystrophic *mdx* mice, the total number of muscle FAPs was reduced, while the proportion of cells expressing PPARγ was increased, suggesting that FAPs are more committed to adipogenic differentiation when exposed to the cell microenvironment of older dystrophic mice (Giuliani et al., 2021).

Aging impairs the paracrine pro-myogenic effect of FAPs to support MuSC activation and myogenesis (Lukjanenko et al., 2019). This effect is mediated by the FAP-derived matricellular protein WISP1. Like the findings of Mozzetta et al. (2013), transplantation of young FAPs or systemic treatment with WISP1 restored the myogenic capacity of MuSCs in aged mice, rescuing muscle regeneration (Lukjanenko et al., 2019). Aging markedly affects FAP proliferation and adipogenic fate in mice, as the total number of FAPs is lower in muscles from aged mice than in those from young mice, and FAPs from aged mice exhibit delayed injury-induced activation and expansion (Lukjanenko et al., 2019). Clonal analysis revealed a reduced capacity for expansion and formation of adipogenic clones, whereas fibrogenic differentiation was enhanced in FAPs from aged mice (Lukjanenko et al., 2019). These changes have been associated with age-related reductions in circulating fibroblast growth factor 2 (FGF2) and the SPARC myokine (*Osteonectin*) (Mathes et al., 2021). In this context, MuSCs and mature myofibers exert juxtacrine anti-adipogenic effects on FAPs via the FGF2/miR-29a/SPARC signaling pathway (Mathes et al., 2021). FGF2 is actively secreted by FAPs, MuSCs and myofibers. This growth factor signals through the FGF receptor to activate MEK1/2 and ERK1/2, which upregulate *miR-29a* expression. Elevated miR-29a stimulates FAP adipogenesis through a reduction in SPARC, promoting IMAT formation (Mathes et al., 2021). Taken together, these findings support the idea that reduced muscle regeneration in aged muscles could be due to age-related changes in FAP chromatin dynamics, function and fate.

A recent study showed that atrophic muscles in aged mice were characterized by upregulation of hallmarks of senescence, including the cyclin-dependent kinase inhibitors *p16* and *p21*, together with telomere dysfunction, and loss of nuclear high mobility group box 1 protein (HMGB1) and Lamin B1 (Zhang et al., 2022). Interestingly, FAPs were the most predominant cells expressing *p16.* Senescent p16^high^ FAPs showed upregulation of senescence-related genes and processes including proliferation regulation, collagen processing, chemokine signaling, cytokine-cytokine receptor interaction, and MAK signaling (Zhang et al., 2022). Importantly, treatment with senolytic agents dasatinib and quercetin markedly attenuated the expression of senescence-related genes and pathways in skeletal muscle of old mice compared to untreated old counterparts and young mice (Zhang et al., 2022). Overall, these findings highlight a pathogenic role of senescent FAPs in age-related muscle sarcopenia.

### 5.8. FAP adipogenesis in rotator cuff tears

One key physiopathological example of FAP activation and proliferation and subsequent IMAT formation in musculoskeletal disorders is the extensive fibro-fatty infiltration of rotator cuff muscles after tearing (Liu et al., 2016). IMAT in response to rotator cuff tears (RCTs) compromises muscle-tendon repair, leading to poor functional outcomes, muscle atrophy, and tendon retearing (Goutallier et al., 2003; Gladstone et al., 2007; Klatte-Schulz et al., 2014). Following RCT, FAPs proliferate and undergo fibro-adipogenesis leading to rapid accumulation of fibrosis and IMAT near the site of the tendon tear. Here, IMAT is formed by a high number of small FAP-derived adipocytes clustering in large fat clumps rather than as enlarged hypertrophic adipocytes (Trudel et al., 2019). This adipocyte hyperplasia indicates continuous stimulation of adipogenesis in FAPs throughout the progression of muscle degeneration (Trudel et al., 2019). The association between IMAT and rotator cuff muscle atrophy may be related to the FAP-derived adipocyte secretome, which includes extracellular vesicles carrying microRNAs, fatty acids (FAs), and pro-inflammatory cytokines that can interfere with muscle signaling and metabolic pathways associated with muscle regeneration (Sachs et al., 2019; Sandona et al., 2020). This highlights the pathogenic role of FAP-derived adipocytes in muscle atrophy following RCT. Interestingly, treatment with retinoic acid receptor agonists (Shirasawa et al., 2021), inhibition of PDGFR signaling by imatinib (Shirasawa et al., 2017), the TGF-βR small molecule inhibitor SB431542 (Davies et al., 2016), and GDF10/BMP-3b inhibitors reduce FAP adipogenesis and muscle atrophy in animal models of RCTs, opening up new therapeutic possibilities for debilitating RCTs.

Although the aberrant behavior of FAPs in chronic muscle damage and myopathy suggests that they play a major role in the pathogenesis of these diseases (Uezumi et al. 2010, 2021; Mozzetta et al., 2013; Buras et al., 2019; Hogarth et al., 2019; Eisner et al., 2020; Farup et al., 2021), this relationship is not necessarily causative. The associations can be divided into at least two components. First, chronic cycles of degeneration and regeneration can lead to abnormal FAP behavior, regardless of the initial trigger; second, after the FAP steady state is disrupted and fibro-adipogenesis is unleashed, this altered behavior can further contribute to the progression and worsening of subsequent pathologies. A better understanding of these components may shed light on the development and potential treatment of pathologies associated with aberrant FAP behavior.

### 5.9. Role of hypoxia and vascularization in FAP adipogenesis

#### 5.9.1. Hypoxia inducible family (HIF) of transcription factors

Hypoxia refers to physiological or pathophysiological-induced states in which normal oxygen tension is reduced. The molecular and cellular adaptations to hypoxia are primarily mediated by the hypoxia inducible family (HIF) of the transcription factors alpha and beta (HIF-α and HIF-β). Under normal oxygen concentrations, HIF prolyl hydroxylase enzymes hydroxylate HIF-α subunits at conserved proline residues, which are recognized and ubiquitinated via the Von Hippel–Lindau E3 ubiquitin ligase and thus targeted for proteasomal degradation. However, hypoxia usually leads to the stabilization of HIF-α subunits via a mechanism involving low-oxygen-driven inhibition of the HIF prolyl-hydroxylase-Von Hippel‒Lindau E3 ubiquitin ligase axis (Schofield and Ratcliffe 2004; Lee et al., 2020d). On the other hand, hypoxia blocks degradation and stabilizes HIF-α, which allows its cytoplasmic accumulation and translocation to the nucleus, where it heterodimerizes with one constitutively expressed HIF-β subunit to transactivate genes involved in the adaptation and sensing of hypoxia (Choudhry and Harris 2018).

HIF-1α and HIF-2α are essential for adult muscle regeneration but dispensable for embryonic skeletal muscle development (Yang et al., 2017). Acute injury induces hypoxia and inhibits FAP adipogenesis (Drouin et al., 2019) by disrupting the vascular network (Morton et al., 2019). Furthermore, hypobaric hypoxia compromises skeletal muscle regeneration after notexin-induced damage in rats (Chaillou et al., 2014). Muscle hypoxia-like states are also observed after denervation in ALS-affected hSOD1^G93A^ mutant mice and dystrophic *mdx* mice and are correlated with reduced endothelial cell density (Valle-Tenney et al., 2020). However, bulk HIF-1α protein levels vary in these models of muscle disease (Valle-Tenney et al., 2020), suggesting differential regulation of HIF-1α in distinct myo-degenerative conditions. Increased HIF expression was linked to IMAT and atrophy after tendon retraction in humans (Lakemeier et al., 2010), where HIF expression was proportional to the magnitude and extent of damage. On the other hand, Lee et al. (2017b) showed that hypoxia increases HIF-1α and HIF-1β binding to the FABP4 promoter in C3H10T1/2 cells, leading to increased FAPB4 mRNA and protein levels (Lee et al., 2017b). Complete supraspinatus tendon transection, a mouse model of RCT, is related to hypoxia-induced IMAT accumulation, which is reversed by treatment with either HIF-1 or FABP4 inhibitors (Lee et al., 2017b). These findings contrast with the hypoxia-driven anti-adipogenic effects of FAPs (Drouin et al., 2019) and reveal the association of fat infiltration after RCT with hypoxia-mediated HIF induction and its effect on adipogenic FABP4 expression (Lee et al., 2017b).

A recent preprint revealed that muscle FAPs expressed more HIF-1α than MuSCs did, and CTX injury-induced hypoxia promoted the proliferation of FAPs through a mechanism mediated by mTORC and HIF-1α (Ollitrault et al., 2021). The authors also confirmed that hypoxia blocks FAP adipogenesis, as previously described (Drouin et al., 2019). These findings could explain the environment-driven differentiation of FAPs toward an activated fibroblast rather than an adipogenic phenotype upon acute injury-induced hypoxia. We recently explored the role of HIF-1α in cardiac fibroblasts (likely FAPs) in response to myocardial infarction. These cells had an elevated hypoxic status together with increased levels of HIF-1α compared with those in the total interstitial cell fraction (Janbandhu et al., 2022). Conditional knockout of *Hif-1α* enhances the proliferation of PDGFRα^+^ cells postcardiac injury, likely driven by priming cell cycle entry after myocardial infarction (Janbandhu et al., 2022). These findings indicated that tissue challenges, hypoxia, and disease conditions play pivotal roles in controlling the behavior and dynamics of fibroblast lineages. Potential therapeutic interventions utilizing oxygen tension and the HIF axis in diseased muscles have yet to be demonstrated.

#### 5.9.2. Vascular endothelial growth factor a (VEGFA)

A recent study revealed that FAPs, immune cells, and MuSCs secrete the pro-angiogenic factor VEGFA (Groppa et al., 2023). First, hematopoietic cell-derived VEGFA is essential for macrophage infiltration and vascular remodeling in response to damage; therefore, immune-derived VEGFA is critical for muscle regeneration (Groppa et al., 2023). On the one hand, MuSC-derived but not FAP-derived VEGFA was necessary for efficient muscle regeneration following notexin-induced damage (Groppa et al., 2023). On the other hand, following femoral artery ligation to induce ischemic damage, VEGFA secreted by FAPs is critical for inducing endothelial cell proliferation and angiogenesis. Conversely, the lack of this signal induces a pro-inflammatory phenotype, mostly via IL-6 upregulation in FAPs. Notably, FAP adipogenesis was upregulated in the absence of MuSC-derived VEGFA but markedly reduced in mice lacking FAP-derived VEGFA, demonstrating an autocrine anti-adipogenic effect of FAP-derived IL-6 secretion (Groppa et al., 2023). Ischemic damage to skeletal muscle reorganizes the basement membrane, an ECM-based physical barrier that separates MuSCs, endothelial cells, and FAPs into different compartments within the interstitial space and myofibers. Ischemia-induced basement membrane rupture allows FAPs and endothelial cells to interact. This reorganization allows FAP-derived VEGFA to be delivered directly to endothelial cells, thus providing the required signal for EC proliferation and angiogenesis (Groppa et al., 2023).

Aging alters VEGFA signaling in muscles. Compared with young mice, aged mice have reduced levels of VEGFA in skeletal muscle and an impaired regeneration capacity after injury (Endo et al., 2023). This effect can be rescued via systemic administration of the small molecule ML228, which induces HIF-1α nuclear translocation and stimulates VEGF production (Endo et al., 2023). Genetically mediated global reduction in *Vegfa* (probably affecting global VEGFA production by different cell types, including immune cells, MPs and FAPs) led to marked impairment of regeneration and a threefold increase in IMAT deposition in mice (Endo et al., 2023). This finding suggested compromised crosstalk of VEGFA between different muscle compartments within muscle tissue. Overall, these findings revealed a VEGF-mediated intricate and promiscuous intercellular crosstalk network that regulates vascular remodeling and myogenesis, as well as FAP activation, proliferation, and fate.

## 6. Reduced muscle substrate buffering capacity as a driver of FAP adipogenesis and IMAT: a hypothetical framework

Given that IMAT is a fat depot, it is susceptible to alterations based on an individual’s dietary intake. Daily macronutrient and alcohol intake can influence body fat distribution and the IMAT content in young and old individuals (Sjöholm et al., 2016). Protein and alcohol intake, as well as serum cholesterol levels, were correlated positively with IMAT, whereas carbohydrate intake exerted the opposite effect (Sjöholm et al., 2016). In line with these findings, a recent multi-ethnic study showed that heavy alcohol intake and binge alcohol consumption significantly increase the accumulation of ectopic fat in different tissues, including IMAT (Kazibwe et al., 2023). However, in the case of IMAT, this association was not significant once adjusted for other health-related habits, including physical activity (Kazibwe et al., 2023). Studies using short-term metabolic challenges, such as a HFD *in vivo*, glucose-lowering agents, and fatty acid treatments in FAP cultures, have demonstrated a shift toward a pathological state involving the remodeling of FAP oxidative metabolism (Aguiari et al., 2008; Agley et al., 2013; Buras et al., 2019; Reggio et al., 2020a; Farup et al., 2021). In this section, we elaborate on a hypothetical framework for how reduced muscle activity may impair metabolic flexibility within muscles and discuss the evidence for a link between these phenomena and adipogenesis.

### 6.1. Substrate-driven metabolic reprogramming of FAPs

FAPs respond to metabolic challenges in skeletal muscle by remodeling the metabolic machinery in accordance with the prevailing cellular environment. At rest, FAPs rely mainly on mitochondrial fatty acid (FA) oxidation for energy production (Reggio et al., 2020a). Proteomic profiling of FAPs revealed that proteins involved in key metabolic processes are differentially regulated upon activation and differentiation in muscular dystrophy (Marinkovic et al., 2019; Reggio et al., 2020a). The expression of enzymes involved in FA β-oxidation, the tricarboxylic acid cycle, oxidative phosphorylation and lipid biosynthesis is downregulated, thus reducing oxygen consumption, the mitochondrial potential, and ATP production (Reggio et al., 2020a). Conversely, enzymes that participate in glycolysis and the pentose phosphate cycle, including pyruvate kinase M2 and glucose-6-phosphate dehydrogenase, are upregulated (Marinkovic et al., 2019; Reggio et al., 2020a). These findings are consistent with a metabolic switch of FAPs in a dystrophic milieu, with upregulated glycolytic, anabolic-like metabolism (Reggio et al., 2020a). A reliance on glycolysis, with subsequent increased lactate production, has been reported in human fibrogenic CD90^+^ FAPs (Farup et al., 2021). This metabolic switch was associated with a pro-fibrogenic phenotype required to synthesize ECM (Farup et al., 2021). Interestingly, adipogenic CD90^−^ FAPs also rely on glycolysis when exposed to PDGF-AA (Farup et al., 2021). In line with these findings, an increase in glycolytic flux was associated with increased proliferation and adipogenic differentiation of FAPs isolated from the muscles of *mdx* mice (Reggio et al., 2020a). Interestingly, a short-term HFD regimen restored the expression of mitochondrial proteins; rescued FA oxidation and oxygen consumption; and increased ATP production while reducing the proliferation of dystrophic FAPs. Together, these effects ameliorate the dystrophic muscle phenotype in mice (Reggio et al., 2020a).

High glucose reprograms the adipogenic potential of precursor cells by upregulating adipogenic transcription factor expression and inhibiting anti-adipogenic WNT/β-catenin signaling (Keats et al., 2014; Kolodziej et al., 2019). This effect may be mediated by the production of ROS and subsequent activation of conventional protein kinase C-beta (PKCβ) (Aguiari et al., 2008). PKCβ synergistically potentiates PPARγ transcriptional activity during adipogenesis (Zhou et al., 2006), which likely explains these effects.

Cardiac FAPs exhibit increased glycolytic metabolism in association with increased proliferation, scar formation and reactive oxygen species (ROS) release, leading to contractile dysfunction after myocardial infarction (Janbandhu et al., 2022). Importantly, by fractionating cardiac FAPs based on mitochondrial mass, we reported that Mito^high^ FAPs have greater fibrogenesis potential but lacks adipogenic potential *in vitro*. In contrast, ~15% of the Mito^low^ and Mito^medium^ FAP fractions differentiated into PLIN1^+^ adipocytes upon adipogenic stimulation, indicating that FAPs are metabolically heterogeneous, with progenitor cells maintaining a lower mitochondrial mass, and reinforcing the notion that mitochondrial dysfunction could drive the adipogenic fate of FAPs (Janbandhu et al., 2022) (Fig. 4D).

In individuals with T2DM, both arterial and interstitial glucose concentrations are increased, reducing the transcapillary glucose gradient (Frossard et al., 2005). This can be attributed to reduced muscle insulin signaling transduction, which leads to insulin resistance and reduced insulin-stimulated glucose uptake (~30–40%) in individuals with T2DM (Sjöstrand et al., 2000; Middelbeek et al., 2013). Taken together, these findings suggest that impaired insulin signaling transduction and reduced local blood flow may increase muscle interstitial glucose and insulin concentrations, resulting in their accumulation in the muscle interstitium. This could affect FAP differentiation capacity, particularly in FAP subtypes enriched in lipid and adipogenesis pathways, such as FBN1 FAPs in humans and Prg4 FAPs in mice (Rubenstein et al., 2020; Yang et al., 2021). Insulin is the primary hormonal regulator of adipogenesis in progenitor cells and preadipocytes (Czech et al., 2013) and of lipid turnover in mature adipocytes (Shao et al., 2016; Krycer et al., 2020). In patients with diabetes, a larger arterial-interstitial insulin gradient and transcapillary transport time are required for insulin to reach and stimulate glucose uptake (Sandqvist et al., 2011). The accumulation of IMAT has been suggested to impair insulin diffusion through the muscle interstitial space, leading to insulin resistance in dogs (Kolka et al., 2010). These findings suggest that reduced insulin clearance from the interstitial space under metabolic conditions may generate proper medium for stimulating FAP adipogenesis. In addition to glucose serving as a source for de novo lipogenesis, lactate can also be utilized to synthesize glycerol-3-phosphate and increase TG synthesis, thus supporting the storage of excess energy in conditions of impaired glucose metabolism (Nye et al. 2008a, 2008b). Plasma lactate is increased in patients with obesity, metabolic syndrome, or T2DM and is inversely correlated with insulin sensitivity (reviewed by (Broskey et al., 2020)). An increased interstitial lactate concentration can modulate vascular tone in muscle arterioles, reducing muscle blood flow and preventing lactate clearance from the interstitial space (Qvisth et al., 2007). This interstitial lactate may be taken up by FAP-derived adipocytes and utilized as a lipogenic precursor. Muscle-derived lactate stimulates TGF-β2 expression in adipocytes (Takahashi et al., 2019). High levels of lactate in the muscle interstitial space can induce the expression and secretion of TGF-β2 from FAPs, favoring their proliferation and survival. Overall, these findings suggest that impaired glucose metabolism within muscle, characterized by increased interstitial levels of glucose, lactate, and insulin, may provide a favorable cellular environment for the adipogenic differentiation of FAPs.

Long-chain fatty acids are ligands of PPARγ and can induce adipogenesis of precursor cells in adipose tissue (Amri et al., 1995; Shao et al., 2016). Physiological doses of FAs increase the intracellular lipid content and upregulate the expression of *Pparγ* and *C/ebpα* in both human FAPs and MuSCs in culture, but adipocyte formation occurs only in FAPs (Agley et al., 2013). A 4-week short-term HFD intervention improved mitochondrial function and FA oxidation in muscular dystrophy FAPs and upregulated anti-adipogenic WNT/β-catenin signaling and the expression of the pro-myogenic factor *follistatin*. Overall, these changes led to an improved dystrophic phenotype in adult *mdx* mice (Reggio et al., 2020a).

Long-term (20-week) HFD-induced obesity led to interstitial accumulation of the lectin family protein galectin-3 (Gal-3), a potent chemoattractant (Takada et al., 2022) involved in cell adhesion, cell cycle progression, apoptosis, inflammation, cell proliferation, and differentiation (Henderson and Sethi 2009). Gal-3 is predominantly secreted by immune cells, mature adipocytes, and stromal cells in visceral and subcutaneous adipose tissues, and its expression is upregulated in individuals with obesity and T2DM (Weigert et al., 2010; Rhodes et al., 2013). Conversely, Gal-3 knockdown reduced adipocyte differentiation via downregulation of the transcriptional activity of PPARγ, C/EBPα, and C/EBPβ and the expression of lipogenic genes in visceral adipose tissue and the liver (Kiwaki et al., 2007; Baek et al., 2015).

A chronic HFD has been shown to increase FAP adipogenesis and muscle dysfunction (Buras et al., 2019; White et al., 2021; Zhang et al., 2021). Likewise, a 20-week HFD induced FAP adipogenic differentiation and IMAT accumulation in hindlimb muscles, with more accumulation in the quadriceps than in the gastrocnemius and *tibialis anterior* muscles (Takada et al., 2022). Moreover, a HFD impaired muscle regeneration after CTX-induced injury by accelerating IMAT formation and promoting myofiber atrophy; similar responses were observed in genetically obese *ob/ob* and *db/db* mice (Takada et al., 2022). These findings indicate that obesity induces IMAT formation independently of diet composition, similar to what has been described in obese, insulin-resistant *db/db* and KK-A^y^ mice, a genetic model of obesity and diabetes characterized by altered adipokine expression, dyslipidemia, and insulin resistance (Mogi et al., 2016).

Finally, impaired circulating levels of hormones such as insulin, GH, IGF-1, estradiol, and testosterone T3 (Freda et al., 2008; Gill et al., 2016; Farup et al., 2021); various cytokines, including TSP-1, DKK1, CCN1, IL-8, MCP1, TNF-α, FGF2, FGF21 and SPARC (Poehlman et al., 2000; Varma et al., 2008; Cheema et al., 2010; Haam et al., 2016; Ali et al., 2019; Buras et al., 2019; Hong et al., 2019; Hu et al., 2019; Mathes et al., 2021; Yang et al., 2021); circulating TG; lipoproteins; and metabolites such as creatinine and ammonia (Gilsanz et al., 2010; Kitajima et al., 2013; Montano-Loza et al., 2016; Nardelli et al., 2019), have all been associated with increased IMAT content in chronic diseases (Table 1).

## 7. Exercise as a treatment against pathological IMAT accumulation

Investigating the impact of exercise training on ectopic IMAT in adults with chronic diseases, we found that moderate-intensity aerobic exercise, alone or in combination with resistance training, effectively reduced IMAT (Tunon-Suarez et al., 2021). Exercise may affect FAPs and FAP-derived adipocytes, although the mechanisms underlying this effect have not been fully elucidated. For example, exercise can reduce muscle glucose interstitial concentration through contraction-stimulated glucose uptake, independent of insulin-mediated capillary vasodilation (McConell et al., 2020), and downregulate ECM deposition and FAP adipogenic differentiation in insulin-resistant states (Yang et al., 2021). Hence, exercise has the potential to counteract the impact of impaired insulin signaling transduction and diminished local blood flow on interstitial glucose availability. This may lead to a reduction in the adipogenic differentiation capacity of FAPs. In a mouse model of chronic inflammation, exercise training combined with pharmacological activation of AMP-dependent protein kinase prevented muscle fatty degeneration by rescuing apoptosis-mediated clearance of FAPs (Saito et al., 2020). *In vitro* treatment of human pro-adipogenic CD90^−^ FAPs with the exercise mimetic drug metformin completely blunted adipogenesis (Farup et al., 2021). Notably, bona fide myokines such as IL-6 and IL-15 (Kang et al., 2018; Madaro et al., 2018) and other factors associated with exercise responses, including histamine and nitric oxide (Cordani et al., 2014; Kasai et al., 2017), are involved in the regulation of FAP proliferation and fat cell differentiation. Although these findings have not been described in the context of exercise, they suggest potential effects of the milieu and circulating factors produced by active muscles (also known as exerkines) on FAP homeostasis and fate. For a review of drug strategies used to target muscle fibro-adipogenic progenitor differentiation and adipogenic differentiation, see (Contreras et al., 2021b; Giuliani et al., 2022).

A recent study analyzed the transcriptomic responses of young mice to 4 weeks of voluntary wheel running exercise and a HFD in subcutaneous and visceral adipose tissues and skeletal muscle (Yang et al., 2022). In these three metabolic tissues, whole-tissue and single-cell transcriptomics revealed cell-state- and cell-type-specific gene expression changes in response to diet-induced obesity with or without exercise training. In skeletal muscle, exercise increased the content of FAPs and myeloid and endothelial cells, and the combined HFD and exercise intervention had a greater effect on these cells. The main effects observed in FAPs were related to the differential expression of genes involved in FA processing, mitochondrial respiration, the antioxidant response, the immune response, the downregulation of ECM remodeling (synthesis, organization) and adipogenic differentiation, and the upregulation of rhythmic processes and circadian behavior. The anti-adipogenic effect was suggested to be related to an increase in CD142^+^ FAPs (Yang et al., 2022).

Exercise training was shown to increase FAP numbers in mouse models of cancer, including rhabdomyosarcoma, preneoplastic colorectal lesions, and radiation-induced muscle damage (Roubos et al., 2021; Collao et al., 2023). Additionally, D’Souza et al. (2019) reported that in irradiated muscles, a HFD reduced the FAP content but increased the level of markers of fibro-fatty infiltration in muscle (D’Souza et al., 2019). Furthermore, exercise training upregulated FAP-tenocyte and FAP-macrophage cross-talk, and the combination of exercise and HFD increased fibrosis and muscle adiposity in irradiated muscles (D’Souza et al., 2019). Together, these findings shed light on the molecular mechanisms underlying the beneficial effects of exercise on IMAT, which can inform the development of exercise-based interventions for disorders characterized by increased IMAT accumulation.

To elucidate the potential effect of exercise on reducing IMAT, studies have quantified the amount of ectopic IMAT in muscles using noninvasive imaging techniques (e.g., computed tomography, magnetic resonance imaging, and ultrasound) (Goodpaster et al., 2008; Cadore et al., 2014; de Almeida et al., 2020). Unfortunately, these methods cannot be used to distinguish between intramyocellular triglycerides and extramyocellular (i.e., IMAT) lipid compartments. Early studies analyzing the effect of intramuscular lipids by biochemical determination of total TG also failed to distinguish intramyocellular lipids from extramyocellular lipids (Pan et al., 1997; Goodpaster et al., 2000a). These technical limitations make it difficult to establish whether the FAs released from contracting muscles into the bloodstream are derived from the IMTG or IMAT pools. Studies combining different methods to measure IMAT, such as muscle biopsy (Pagano et al., 2018; Sachs et al., 2019) and ^1^H-nuclear magnetic resonance (Hasegawa et al. 2015, 2016; Park et al., 2020), are required to elucidate the exercise-induced cellular and molecular mechanisms involved in the reduction in IMAT after exercise training.

## 8. Concluding remarks

FAPs play a crucial role in muscle homeostasis and regeneration. However, their progressive activation, survival, and differentiation also contribute to metabolic disturbances leading to loss of muscle mass, fibro-fatty replacement, IMAT and insulin resistance. In this review, we discussed the current knowledge and highlight research gaps related to the identity, characteristics, and roles of FAPs in the ectopic accumulation of adipocytes and IMAT. The activation, commitment, and terminal differentiation of pro-adipogenic subpopulation(s) of FAPs are consistently regulated by crosstalk mechanisms and substrate availability within the muscle niche. Overall, the recruitment and expansion of FAP subgroups in muscles are not only closely linked but also constantly adapt once homeostasis has been disrupted. This plasticity enables muscles to adjust to metabolic and biomechanical disruptions. However, the available evidence strongly suggests that the muscle environment, immune milieu, and developmental origin play crucial roles in determining the identity and fate of FAPs. Herein, we propose a mechanism in which reduced muscle contractile activity impairs the capacity of myofibers to buffer energy substrates, leading to cellular metabolic inflexibility, which is sensed by FAPs, after which the cells differentiate into adipocytes. Although this hypothesis highlights the importance of FAPs in the development of ectopic fat accumulation, many questions remain unanswered, including how FAPs sense myofiber contractile activity, and what metabolic cues from surrounding muscle cells are involved. Additionally, the athlete’s paradox, which refers to the similarity of the density of lipid droplets in muscle cells between well-trained individuals and patients with obesity, raises further questions regarding the relationship between exercise, FAPs, and lipid compartmentalization within skeletal muscle. A better understanding of the contribution of FAPs to metabolic disorders will provide insights into new therapeutic strategies for these conditions.

## Figures and Tables

**Table 1:** IMAT accumulation and its relationship with metabolic and functional markers in human chronic diseases, and immunometabolic and musculoskeletal disorders.

Diagnosis	IMAT	IMTG	Lean mass	Association with circulating and functional markers	Reference

**Aging**	↑	?	↓ or =	↓ Insulin sensitivity, ↓ Adiponectin, ↓↑Leptin, ↑ IL-6, ↑ Myostatin, ↑ skeletal muscle fibrosis, mobility limitations, ↑ risk of fall	(Visser et al., 2005; Zoico et al., 2013, Kim, Dunville et al. 2017; Konopka et al., 2018; Yoshiko et al., 2018; Vitale et al., 2021)
**Reduced muscle contractile activity**	↑	↑	↓	↑, = Thigh-SCAT	(Manini et al., 2007, Brooks et al., 2008, Taaffe et al., 2009; Popadic Gacesa, Kozic et al., 2011, Burkhart et al., 2019)
**Hormonal Disorders (acromegaly, PCOS, Post menopause)**	↑, =	↑?	↓, =	↓Insulin sensitivity ↓HDLc, ↑TG, ↓vit-D	(Freda et al., 2008; Freda et al., 2009; Reyes-Vidal et al., 2015; Goss et al., 2014; Scott et al., 2016; Goss et al., 2014; Scott et al., 2016)
**Immunometabolic and cardiovascular disorders**	↑	↑	↓, =	↓ Insulin sensitivity, Dyslipidemia ↓GH, ↑MCP-1, ↑FGF21, ↓FGFR1, ↑adiponectin, ↓Leptin, ↑ creatinine, ↑RCP, ↑ Hypertension Blood pressure, ↑T2DM risk & prevalence, ↑ diabetic peripheral neuropathy	(Yim et al., 2007; Therkelsen et al., 2013) (Hasegawa et al., 2015; Hasegawa et al., 2016; Komiya et al., 2006; Bergia et al., 2018; Lim et al., 2019; Park et al., 2020) (Goodpaster et al., 2023; Boettcher et al., 2009; Register et al., 2013; Hong et al., 2019; Lo et al., 2007; Yoshimura et al., 2011; Yaskolka Meir et al., 2016; Khoja et al., 2018)
**Advanced cardiovascular disease**	↑	↑?	↓, =	↑CMR, ↑ muscle wasting, ↑ RCP, ↑ major cardiovascular events, ↑ coronary artery calcification	(Terry et al., 2017; Matsubara et al., 2018; Sugai et al., 2018; Yamashita et al., 2019)
**Musculoskeletal disorders:** KOA, CLBP, RCT, SCI	↑	↑, =	↓	↑ cartilage loss, ↑sarcopenia & ↓ functional capacity, ↑ muscle PDGFRα/PLIN expression, predictor of disease prognosis	(Ruhdorfer et al., 2015; Teichtahl et al., 2015; Ikemoto-Uezumi et al., 2017; Hebert et al., 2014; Sions et al., 2017; Hebert et al., 2020; Goutallier et al., 2003; Gladstone et al., 2007; Klatte-Schulz et al., 2014; Davies et al., 2022; Davies et al., 2022)
**Muscular dystrophies:** *BMD, DMD, LGMD2A, Sodium channelopathies*	↑↑	?	↓↓	↓ Irisin, ↑ leptin, ↑ FABP4, ↑ NAMPT, ↑ CNDP1, ↓ PLA2G2A, ↓ MYBPC1, ↓ CKM. Myocardial fibrosis and cardiomyopathy	(Leroy-Willig et al., 1997; Gong et al., 2000; Mankodi et al., 2016; Marty et al., 2018; Spitali et al., 2018; Schlaeger et al., 2019, Henson et al., 2021; Sun et al., 2021; Barp et al., 2022)
**Neuromuscular diseases and inflammatory myopathies:**	↑↑	?	↓, =	Progressive functional deficits, ↑ nerve damage & muscle edema, Hypermetabolic state, ↑ resistin, visfatin, PAI-1, GLP1, GIP, C-peptide, adipsin, adiponectin, dyslipidemia, ↑	(Lichtenstein et al., 2018; Kim et al., 2022; Morrow et al., 2016; Cornett et al., 2019; Kim et al., 2019; Diamanti et al., 2019; Hackett et al., 2019; Li et al., 2022; Li et al., 2022; Vernikouskaya et al., 2022; Steyn et al., 2018; Laurent et al., 2022)
**Chronic organ disease:** COPD, CKD, CLD	↑	↑	↓ or =	↓Thiol/protein ratio, ↓Endothelial function, ↑ disease progression and morbidity, ↑IL-8, ↓albumin & prealbumin, ↓phosphates, ↑Amonnia, ↑creatine, ↓insulin sensitivity	(Maddocks et al., 2014; Shields et al., 2015; Coats et al., 2018; Awano et al., 2020; Cheema et al., 2010; Keddar et al., 2020; Kitajima et al., 2013; Montano-Loza et al., 2016; Bhanji et al., 2018; Nardelli et al., 2019)
*Muscle wasting: Cancer, HIV,*	↑, =	?	↓, =	↓ survival, ↑ poor prognosis, ↑ frailty, ↑dyslipidemia, ↓ insulin sensitivity	(Fujiwara et al., 2015; Silva de Paula, de Aguiar Bruno et al. 2018; Williams et al., 2018; Mardian et al., 2019; Albu et al., 2007; Scherzer et al., 2011)
*Anorexia Nervosa*	↓	?	↓	↑ Estradiol	(Mayer et al., 2005; Gill et al., 2016)

HDL-c: high-density lipoprotein cholesterol, TG: triglycerides, MCP-1: monocyte chemoattractant protein-1, FGF21: fibroblast growth factor 21, FGFR1: fibroblast growth factor receptor 1, CLBP: chronic low back pain, CMR: cardiometabolic risk, RCP: reactive C-protein, MCP-1, FGF21, FGFR1, NAMPT: nicotinamide phosphoribosyltransferase, CNDP1: Carnosine dipeptidase 1, PLA2G2A: phospholipase A2 group IIA, MYBPC1: Myosin Binding Protein C1 CKM: muscle creatine kinase.

## Abbreviations

AnxA2: Annexin-A2
C/EBPα: CCAAT/enhancer-binding protein alpha
CTX: Cardiotoxin
Dlk-1: Delta-like Non-Canonical NOTCH Ligand 1
DMD: Duchenne Muscular Dystrophy
Dpp4: dipeptidyl peptidase 4
ECM: Extracellular matrix
FABP4: fatty-acid binding protein 4
FAPs: Fibro-adipogenic progenitor cells
FBN1: fibrillin-1
GLY: Glycerol
GSK: Glycogen synthase kinase
HDAC: Histone deacetylases
HFD: high-fat diet
Hh: Hedgehog
HIC1: Hypermethylated in Cancer protein 1
HIF: Hypoxia inducible family of transcription factors
IMAT: Intermuscular Adipose Tissue
IMTG: Intramyocellular triglycerides
LUM: lumicanMuSCs: Muscle stem cells
NECD: NOTCH extracellular domain
NICD: NOTCH intracellular domain
NOTCH: Neurogenic locus notch homolog protein
Osr1: Odd-Skipped Related Transcription Factor 1
PDGFRα: platelet-derived growth factor receptor alpha
PKCβ: protein kinase C-beta
PLIN-1: perilipin-1
PPARγ: Peroxisome Proliferator-Activated Receptor Gamma
RCT: rotator cuff tear
SCAT: Subcutaneous adipose tissue
sc-RNAseq: single-cell RNA sequencing
T2DM: type 2 diabetes mellitus
Tcf7l2: Transcription factor 7-like 2
TG: triglycerides
TGF-β: transforming growth factor beta
TLE: Groucho/Transducin-Like Enhancer
TNF-α: tumor necrosis factor-alpha Trichostatin A TSA
TSP-1: thrombospondin-1
UCP-1: uncoupling protein 1
VEGFA: Vascular endothelial growth factor A
WISP1: WNT1 Inducible Signaling Pathway Protein 1
WNT: Wingless-type MMTV integration site
